# Long-term survival of mechanically ventilated patients with severe COVID-19: an observational cohort study

**DOI:** 10.1186/s13613-021-00929-y

**Published:** 2021-10-02

**Authors:** Oscar Peñuelas, Laura del Campo-Albendea, Amanda Lesmes González de Aledo, José Manuel Añón, Carmen Rodríguez-Solís, Jordi Mancebo, Paula Vera, Daniel Ballesteros, Jorge Jiménez, Emilio Maseda, Juan Carlos Figueira, Nieves Franco, Ángela Algaba, Juan Pablo Avilés, Ricardo Díaz, Beatriz Abad, Alfonso Canabal, Ana Abella, Federico Gordo, Javier García, Jessica García Suarez, Jamil Cedeño, Basilia Martínez-Palacios, Eva Manteiga, Óscar Martínez, Rafael Blancas, Tommaso Bardi, David Pestaña, José Ángel Lorente, Alfonso Muriel, Andrés Esteban, Fernando Frutos-Vivar

**Affiliations:** 1 Intensive Care Unit, Hospital Universitario de Getafe, Carretera de Toledo km 12,500, 28905 Madrid, Spain; 2 Centro de Investigación Biomédica en Red de Enfermedades Respiratorias, Instituto de Salud Carlos III, Madrid, Spain; 3 Department of Clinical Biostatistics, Hospital Ramón Y Cajal, Instituto Ramón y Cajal de Investigación Sanitaria & Centro de Investigación Biomédica en Red de Epidemiología Y Salud Pública, Madrid, Spain; 4 Intensive Care Unit, Hospital Universitario Doce de Octubre, Madrid, Spain; 5 Intensive Care Unit, Hospital Universitario La Paz, IdiPAZ, Madrid, Spain; 6 Intensive Care Unit, Hospital Universitari Sant Pau, Barcelona, Spain; 7 Intensive Care Unit, Hospital Universitario Puerta de Hierro Majadahonda, Madrid, Spain; 8 Department of Anesthesiology and Critical Care, Hospital Universitario La Paz, Madrid, Spain; 9 Intensive Care Unit, Hospital Universitario de Móstoles, Madrid, Spain; 10 Intensive Care Unit, Hospital Universitario de Torrejón, Madrid, Spain; 11 Intensive Care Unit, Hospital Universitario Severo Ochoa, Leganés, Spain; 12 Intensive Care Unit, Hospital Universitario La Princesa, Madrid, Spain; 13 Intensive Care Unit, Hospital Universitario del Henares, Coslada, Spain; 14 Department of Anesthesiology and Critical Care, Hospital Universitario Puerta de Hierro Majadahonda, Madrid, Spain; 15 Intensive Care Unit, Hospital Universitario Gregorio Marañón, Madrid, Spain; 16 Intensive Care Unit, Hospital Universitario Infanta Cristina, Parla, Madrid, Spain; 17 Intensive Care Unit, Hospital Universitario del Tajo, Aranjuez, Madrid, Spain; 18 Department of Anesthesiology and Critical Care, Hospital Universitario Ramón Y Cajal, Madrid, Spain

**Keywords:** Coronavirus disease 2019, COVID-19, ARDS, Intensive care unit, Invasive mechanical ventilation, Mortality, Pneumonia, SARS-CoV-2, Noninvasive ventilation

## Abstract

**Background:**

Information is lacking regarding long-term survival and predictive factors for mortality in patients with acute hypoxemic respiratory failure due to coronavirus disease 2019 (COVID-19) and undergoing invasive mechanical ventilation. We aimed to estimate 180-day mortality of patients with COVID-19 requiring invasive ventilation, and to develop a predictive model for long-term mortality.

**Methods:**

Retrospective, multicentre, national cohort study between March 8 and April 30, 2020 in 16 intensive care units (ICU) in Spain. Participants were consecutive adults who received invasive mechanical ventilation for COVID-19. Severe acute respiratory syndrome coronavirus 2 (SARS-CoV-2) infection detected in positive testing of a nasopharyngeal sample and confirmed by real time reverse-transcriptase polymerase chain reaction (rt-PCR). The primary outcomes was 180-day survival after hospital admission. Secondary outcomes were length of ICU and hospital stay, and ICU and in-hospital mortality. A predictive model was developed to estimate the probability of 180-day mortality.

**Results:**

868 patients were included (median age, 64 years [interquartile range [IQR], 56–71 years]; 72% male). Severity at ICU admission, estimated by SAPS3, was 56 points [IQR 50–63]. Prior to intubation, 26% received some type of noninvasive respiratory support. The unadjusted overall 180-day survival rates was 59% (95% CI 56–62%). The predictive factors measured during ICU stay, and associated with 180-day mortality were: age [Odds Ratio [OR] per 1-year increase 1.051, 95% CI 1.033–1.068)), SAPS3 (OR per 1-point increase 1.027, 95% CI 1.011–1.044), diabetes (OR 1.546, 95% CI 1.085–2.204), neutrophils to lymphocytes ratio (OR per 1-unit increase 1.008, 95% CI 1.001–1.016), failed attempt of noninvasive positive pressure ventilation prior to orotracheal intubation (OR 1.878 (95% CI 1.124–3.140), use of selective digestive decontamination strategy during ICU stay (OR 0.590 (95% CI 0.358–0.972) and administration of low dosage of corticosteroids (methylprednisolone 1 mg/kg) (OR 2.042 (95% CI 1.205–3.460).

**Conclusion:**

The long-term survival of mechanically ventilated patients with severe COVID-19 reaches more than 50% and may help to provide individualized risk stratification and potential treatments.

*Trial registration*: ClinicalTrials.gov Identifier: *NCT04379258.* Registered 10 April 2020 (retrospectively registered)

**Supplementary Information:**

The online version contains supplementary material available at 10.1186/s13613-021-00929-y.

## Background

The coronavirus disease 2019 (COVID-19) pandemic is one of the most serious health crises in recent decades [1] with over 138 million infections being reported worldwide. As a result, health care resources in many countries are facing unprecedented challenges [2]. This context has been especially dramatic in intensive care units (ICU) in view of the high daily incidence of acute hypoxemic respiratory failure (AHRF) secondary to pneumonia by SARS-CoV-2. Reports show that between 14 and 17% of hospital admissions for COVID-19 require transfer to the intensive care unit (ICU) [3, 4].

Currently available information about critically ill adult patients is heterogeneous because of the case-mix of patients, and in-hospital mortality rates differ between countries [5–9]. Specific data on patient characteristics and long-term survival in critically ill COVID-19 patients are needed to inform decision-making regarding resource allocation, critical care capacity, and therapeutic options. Inter-hospital variation and international clinical variability in treatments and outcomes also needs to be assessed.

We conducted a multicenter cohort study to analyze the long-term mortality of patients who required invasive mechanical ventilation for severe COVID-19 pneumonia, and determine the impact of predictive variables measured during ICU stay for long-term mortality.

## Methods

This observational cohort study was conducted in 16 surgical and medical ICU at 12 university hospitals in Madrid and Barcelona, the Spanish cities most affected by COVID-19 (a complete list of participating sites is provided in the Supplement). We included all patients who were admitted to the participating ICUs between March 8, 2020 and April 30, 2020, and who required invasive mechanical ventilation for acute hypoxemic respiratory failure secondary to SARS-CoV-2 pneumonia. The diagnosis was confirmed by a positive result of real-time reverse-transcriptase polymerase chain reaction (RT-PCR) testing of a nasopharyngeal swab sample or from a lower respiratory tract sample [11]. SARS-CoV-2 pneumonia was also diagnosed when there was a compatible clinical condition, and when typical abnormalities on chest X-ray were present in absence of an alternative diagnosis [10, 11]. Patients were excluded from the analysis if any data regarding relevant variables (Additional file 1: Appendix S1) were missing.

### Data collection

Variables recorded were: age, sex, height, weight, severity at admission estimated by the Simplified Acute Physiology Score (SAPS3) [which ranges from 16 points (low severity) to 226 points (high severity)], comorbidities, (previous medication use), mode of respiratory support (high flow nasal oxygen cannula and/or noninvasive positive pressure ventilation [NPPV] as CPAP (continuous positive airway pressure) or BiPAP (Bi-Level Positive Airway Pressure)), use of compassionate medication [antivirals (lopinavir–ritonavir, remdesivir), hydroxychloroquine, and immunomodulatory agents (interleukin-6 receptor antagonists, Janus kinase inhibitor, and corticosteroids)] before admission to ICU, length of hospital stay prior to ICU admission, date of intubation, ventilatory settings within the first week of mechanical ventilation, daily arterial blood gases, concentrations of plasma/serum biomarkers drawn within 7 days of ICU admission, including high-sensitivity C-reactive protein, D-dimer, ferritin, high-sensitivity troponin, procalcitonin, and IL-6, use of adjuvant therapies for acute respiratory failure (neuromuscular blocking agents, inhaled pulmonary vasodilators, ventilation in prone position, and extracorporeal membrane oxygenation), vasopressor agents, renal replacement therapy, antibacterial agents, antiviral agents, and other immunomodulatory agents, complications, and organ failure during the ICU stay.

Patients were followed-up until 180 days after hospital admission. Patients who died were censored at the date of death for the time-to-discharge analysis.

Independent on-site monitoring was performed at each participating center in coordination with the main investigators who verified all data.

All data were monitored and reviewed by four external investigators (OP, AE, CRS, and FFV) to detect erroneous and missing data.

The study was approved by the institutional ethics board at each participating site. The requirement for informed consent from individual patients was waived, because the study design was considered minimal-risk research using data collected for routine clinical practice during an ongoing public health emergency.

The study was registered in ClinicalTrials.gov Identifier: *NCT04379258*. We followed the Strengthening the Reporting of Observational Studies in Epidemiology (STROBE) statement guidelines for observational cohort studies [12].

### Statistical analysis

Following the approval of the ethics committee at each participating center, patients were collected retrospectively to allow rapid data collection and over 180 days of prospective follow-up to analyze clinical outcomes (data extraction date: April 16, 2020).

The main outcome was mortality at 180 days. Secondary outcomes were duration of mechanical ventilation, duration of ICU and in-hospital stay, reason for death and destiny at hospital discharge.

Variables included in the predictive model were as follows: age, sex, comorbidities (diabetes, hypertension, obesity), and the following pre-admission medications: angiotensin-converting enzyme inhibitors, angiotensin receptor blockers and chronic steroids. We also included in the model non-invasive respiratory support before orotracheal intubation, time from hospital admission until intubation, SAPS3, relevant variables determined within the first 48 h of admission to the ICU (neutrophil to lymphocyte ratio, PaO_2_ to FiO_2_ ratio, D-dimer, ventilatory ratio [14], tidal volume and positive end-expiratory pressure (PEEP)). We also included treatments given within in the first 48 h of mechanical ventilation (vasopressor support, antivirals, immunomodulatory therapy, steroids, anticoagulants, neuromuscular blocking agents, selective digestive decontamination, and prone position). All of the variables were selected by clinical criteria.

Missing data were assumed to be MAR. All missing data were multiply imputed via the Multivariate Imputation by Chained Equations (MICE) procedure [13]. Incomplete dichotomous variable was imputed using a logistic regression model, while linear regression was used to impute incomplete continuous variables. We generated 10 imputed data sets. Since MAR and MNAR cannot be distinguished from observed data, additional sensitivity analyses [14] is shown in the supplement (Additional file 1: Table S10).

Mortality outcomes were analyzed using mixed-effects logistic regression in the imputed data sets. The analysis was fitted with a random effect in which the patients were nested in ICUs to characterize ICU-level variation and to estimate center-specific rates of ICU-mortality. The fractional polynomial method was used to explore the behavior of continuous variables. We applied stepwise selection with backward elimination of predictors from the full model with *p* < 0.05.

The final model was then validated by bootstrapping. Using this procedure we sampled 100 samples for each imputed data set with replacement. Discrimination of the model was evaluated using the *c-statistic*: for a binary outcome, *c* is identical to the area under the receiver operating curve (AUROC). Calibration was evaluated studying the calibration slope and calibration-in-the-large (CITL). This approach indicates the difference between the observed prevalence of death and the predicted mean probability. In a perfect model, the CITL should be equal to zero, but our CITL value was small and the confidence interval contained 0.

The calibration slope tells us if the model coefficients are underfitted (slope > 1) or overfitted (slope < 1). In our case, the slope was below 1, so it was insufficient to obtain the data from our model. However, this is not entirely negative, because as a predictive model, we want the model to be able to explain future data from other patients and units, not just our data. Furthermore, 1 is included in the confidence interval, so we can consider it to be a good model in terms of calibration. [15].

Subsequently, coefficients were used to generate a nomogram to predict the adjusted probability of ICU mortality individually.

To account for differences in patient-level characteristics and illness severity between ICUs, we calculated the median odds ratio (MOR) [16].

Finally, in a sensitivity analysis for the multilevel model, further adjustments were made for the number of ICU beds at each hospital before the COVID-19 pandemic (≤ 50, and > 50 ICU beds).

The 180-day survival outcomes were calculated using a Kaplan–Meier survival plot in an overall cohort and by predefined subgroups (age categorized in ≤ 70 or > 70 years; SAPS3 categorized in ≤ 50 or > 50 points; ratio PaO_2_/FiO_2_ categorized according to the acute respiratory distress syndrome (ARDS) Berlin definition [17]).

The differences between survival curves were evaluated by log-rank test. To identify the optimal cut-point value in ROC analysis we used the Youden index approach. Statistical significance was considered at *p* < 0.05.

Analyses were performed using Stata, version 16.1 (StataCorp LLC).

## Results

### Description of the population included in the analysis

During the study period, 868 of the 1,170 patients who were admitted to the participating ICUs were included in the analysis. A flow chart of study patients is provided in Additional file 1: Figure S1 and clinical characteristics of excluded patients are summarized in Additional file 1: Table S1. Patients’ baseline patient characteristics are shown in Table 1. Seventy-two per cent were males and mean (standard deviation [SD]) age was 62 years [11 years]. The median number of days from onset of symptoms to admission to hospital was 7 days (4, 9 days; p25, p75) and the median length of stay in the hospital before ICU admission was 3 days (1, 5 days; p25, p75). The most common comorbidity was cardiovascular disease (49%). During ward stay before ICU admission, most patients (75%) received some antiviral and/or immune-modulatory therapy, and noninvasive respiratory support was attempted before orotracheal intubation was initiated in 28% of patients (cannula nasal high-flow oxygen in 14% and NPPV in 14%), while 68% received conventional oxygen by venturi or reservoir mask, and 4% received a combination of cannula nasal high-flow oxygen and NPPV.

**Table 1:** Baseline characteristics of included patients in the analysis

	Overall *N* = 868	Survivors *N* = 533	Non-Survivors *N* = 335	*p*-value
Age, mean (SD), years	64 (11)	59 (12)	66 (10)	< 0.001
Female sex	243 (28)	134 (30.8)	79 (23.6)	0.013
Body mass index, mean (SD), kg/cm^2^	29 (5)	29 (5)	29 (5)	0.317
SAPS3, mean (SD), points	57.2 (11.0)	55.2 (9.8)	60.2 (12.1)	< 0.001
Comorbidities				
Hypertension	401 (46)	223 (42)	178 (53)	0.001
Obesity	268 (31)	176 (33)	92 (27)	0.049
Diabetes	209 (24)	103 (19)	106 (32)	< 0.001
Previous therapy				
Angiotensin-converting enzyme inhibitors	172 (20)	95 (18)	77 (23)	0.039
Angiotensin II receptor blockers	122 (15)	69 (13)	53 (16)	0.139
Steroids	34 (4)	15 (3)	19 (6)	0.028
Days from initiation symptoms to admission at hospital, median (P25, P75)	7 (4, 9)	7 (5, 9)	6 (4, 8)	0.078
Days from admission at hospital until orotracheal intubation, median (P25, P75)	3 (2, 6)	3 (1, 5)	3 (1, 7)	0.001
Noninvasive Respiratory support at ward				0.066
Oxygen mask alone	644 (74)	421 (79)	223 (67)	
High flow oxygen nasal cannula alone	105 (12)	66 (12)	39 (12)	
Non-invasive positive pressure ventilation alone	98 (11)	54 (10.1)	44 (13)	
Non-invasive positive pressure ventilation and High flow oxygen nasal cannula	21 (2)	10 (2)	11 (3)	
Ventilatory management at ICU admission				
Tidal volume, mean (SD), ml/kg PBW	7.2 (1)	7.2 (1)	7.2 (2)	0.476
PEEP, mean (SD), cm of water	13 (3)	13 (3)	13 (3)	0.407
Ratio PaO_2_/FiO_2_, mean (SD)	105 (48)	108 (47)	100 (48)	0.016
D-dimer, median (P_25_, P_75_), mg/ml,	3.5 (1, 17)	4 (1, 20)	3.5 (1, 12)	0.425
Ventilatory ratio, mean (SD)	2.1 (0.8)	2.0 (0.8)	2.2 (0.9)	0.009
Ratio neutrophil: lymphocyte, median (P_25_, P_75_)	13 (7, 22)	11 (7, 18)	16 (9, 28)	0.428
Selective Digestive Decontamination	450 (52)	301 (56)	149 (45)	< 0.001
Immunomodulator therapy	347 (40)	210 (39)	137 (41)	0.357
Steroids				0.104
No	449 (52)	276 (52)	173 (52)	
Methylprednisolone ≤ 1 mg/kg	206 (24)	137 (26)	69 (21)	
Methylprednisolone > 1 mg/kg	213 (24)	120 (22)	93 (28)	
Compassionate therapies received during hospital stay				0.013
None	155 (17.9)	89 (16.7)	66 (19.7)	
Antiviral therapy only	196 (22.6)	136 (25.5)	60 (17.9)	
Immunomodulatory agents only	71 (8.2)	35 (6.6)	36 (10.8)	
Antiviral and inmunomodulatory therapies	446 (51.4)	273 (51.2)	173 (51.6)	
Antiviral therapy	796 (92)	492 (92)	304 (91)	0.245
Anticoagulation therapy	90 (10)	55 (10)	35 (10)	0.342
Norepinephrine^a^				< 0.001
No	306 (32)	203 (38)	103 (31)	
Low doses	105 (12)	44 (8)	61 (18)	
High doses	457 (53)	286 (54)	171 (51)	
Days until intubation, median (P_25_, P_75_)	3 (1, 6)	3 (1, 5)	3 (1, 7)	0.002
Meet ARDS criteria at ICU admission				
At ICU admission				0.009
On day 2 from starting ventilatory support				< 0.001
Mild ARDS				
At ICU admission	220 (25.4)	136 (25.5)	84 (25.1)	
On day 2 from starting ventilatory support	331 (38.2)	232 (43.6)	99 (29.6)	
Moderate ARDS				
At t ICU admission	409 (47.1)	267 (50.1)	142 (42.4)	
On day 2 from starting ventilatory support	410 (47.3)	239 (44.9)	171 (51.2)	
Severe ARDS				
At ICU admission	239 (27.5)	130 (24.4)	109 (32.5)	
On day 2 from starting ventilatory support	125 (14.4)	61 (11.5)	64 (19.2)	

Data are *n* (%), unless otherwise indicated

Severity at admission to the ICU estimated by SAPS3 was a mean 57 points (SD, 11 points) (predicted mortality was 29%). At admission to the ICU, serum biomarkers and ventilatory settings, arterial blood gases, adjuvant therapies within the first week of mechanical ventilation (Additional file 1: Table S3 and Figure S2). According to ARDS Berlin criteria, 231 patients (27%) had severe ARDS when mechanical ventilation was started.

Severe ARDS criteria and hypercapnia were also higher in non-survivors: (61% vs. 53%; *p* = 0.018) and (mean PaCO_2_ 56 mmHg vs. 52 mmHg; *p* < 0.001), respectively. Within the first 48 h, ventilatory management (tidal volume, PEEP) was similar in both cohorts (Additional file 1: Table S1 and Figure S2). Early use of ventilatory settings and adjuvant therapies within the first week of ICU stay (prone position, ECMO, nitric oxide inhaled, and neuromuscular blockers) were not different in survivors and non-survivors (Additional file 1: Table S4).

In addition, level of serum biomarkers, and complications within the first week of ICU stay are shown in Additional file 1: Tables S5, S6, S7, Figures S2 and S3.

The neutrophil to lymphocyte ratio was the most relevant biomarker and most significant difference in serum biomarkers (Additional file 1: Tables S1 and S8).

Of the pharmacological treatments initiated in the ICU within the first 48 h, only the implementation of selective digestive decontamination strategy was associated with a significant decrease in mortality (Additional file 1: Table S1).

### Analysis of long-term mortality

**Fig. 1:**
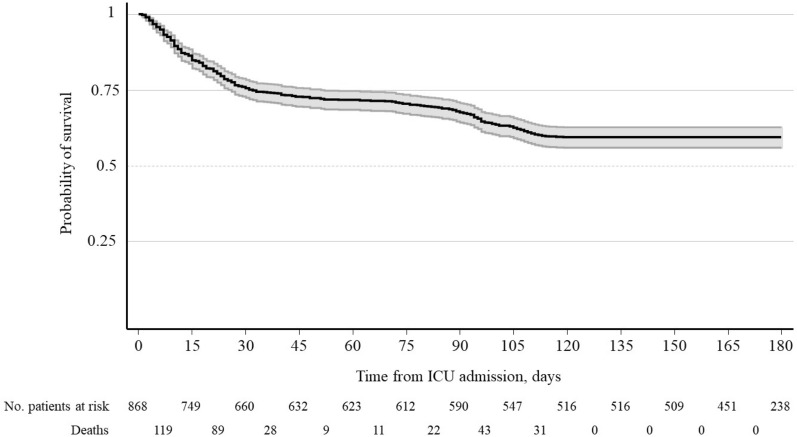
Kaplan–Meier survival curves. Overall survival at 180 days. Grey lines represent the 95% confidence interval

The overall 180-day survival was 59.5% (95% CI 56.1–62.6%) (Fig. 1). Unadjusted Kaplan–Meier survival curves by subgroups showed that survival rates at 180 days of follow-up were lower in patients over 70 years (40% vs. 66.5%, log rank test < 0.001, respectively) (Additional file 1: Figure S4).

**Fig. 2:**
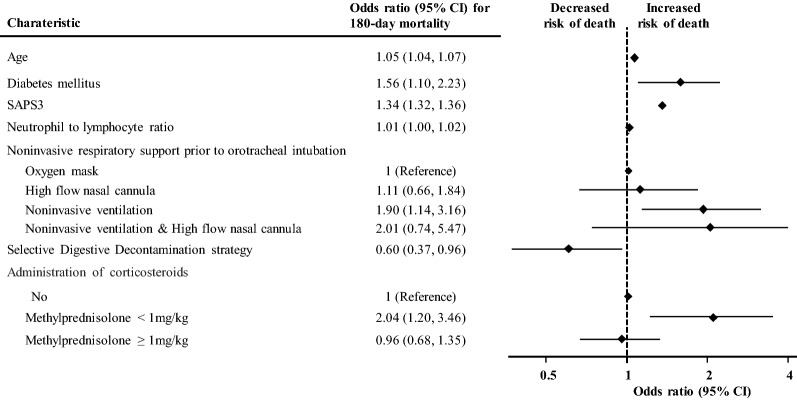
Multivariable-adjusted risk model for death at 180 days and forest plot

Twenty-two variables measured at ICU admission (Additional file 1: Table S8) were included in the multivariable logistic model. In the final multivariable multiple imputation model, after the backwards strategy, the adjusted predictive variables associated with 180-day mortality were: age ( Odds ratio [OR] per 1-year increase 1.051, 95% confidence interval [CI] 1.033–1.068), SAPS3 (OR per 1-point increase 1.027, 95% CI 1.011–1.044], diabetes (OR 1.546, 95% CI 1.085–2.204), neutrophils to lymphocytes ratio (OR per 1-unit increase 1.008, 95% CI 1.001–1.016), failure of noninvasive positive pressure ventilation prior to orotracheal intubation (OR 1.878 (95% CI 1.124–3.140), use of selective digestive decontamination strategy during ICU stay (OR 0.590 (95% CI 0.358–0.972) and administration of low dosage of corticosteroids (methylprednisolone < 1 mg/kg) (OR 2.042 (95% CI 1.205–3.460). The model was internally validated by bootstrapping (Fig. 2). The algorithm had an AUC of 0.716 (95% CI 0.682–0.749), and a calibration, CITL 0.001 (95% CI -0.318–0.307) with slope 0.925 (95% CI 0.734–1.116).

The results of the mixed-effect analysis (Additional file 1: Table S10) showed that center had an effect associated with 180-mortality (median odds ratio [MOR] = 1.829). Sensitivity analysis also showed effects when comparing centers that included more than 50 patients (*N* = 7) with those with less than 50 patients (*N* = 9) and varied widely across centers (Additional file 1: Figure S5).

Concerning survivors at hospital discharge, 426/553 patients were transferred home (77%) and 23% (129/553) were transferred to a nursing facility.

### Other Clinical Outcomes

At the end of ICU stay follow-up (median, 27 days; IQR, 13–46 days). ICU mortality was 38.5% (95% CI 35%–42%).

Among patients discharged alive from the ICU admission, the median ICU length of stay was 17 days (IQR, 10–34 days) and the median hospital length of stay was 38 days (IQR, 26–65 days).

Predictive interpretations compared with 180-day mortality were largely unchanged when restricted to ICU mortality or hospital mortality (Table 2), although the need of vasopressor support was an independent predictor factor for ICU and hospital mortality (OR 1.878, 95% CI 1.119–3-152; OR 2.042, 95% CI 1.205–3.460, respectively).

**Table 2:** Multivariable risk-adjusted predictive model for short- and long-term mortality

Variables	ICU Mortality OR (95% CI)	Hospital Mortality OR (95% CI)	28-day Mortality OR (95% CI)
Age, (per 1-year increase)	1.049 (1.032–1.066)	1.050 (1.033–1.068)	1.058 (1.035–1.081)
Male			
Diabetes mellitus		1.546 (1.084–2.203)	1.626 (1.093–2.420)
Chronic steroids			3.697 (1.633–8.371)
SAPS3, (per 1-point increase)	1.025 (1.008–1.041)	1.027 (1.011–1.044)	
N:L ratio (per 1-unit increase)	1.009 (1.002–1.016)	1.008 (1.000–1.016)	
Failed attempt of NPPV prior to orotracheal intubation	2.131 (1.279–3.550)	1.878 (1.123–3.39)	
Selective Digestive Decontamination strategy	0.587 (0.358–0.963)	0.591 (0.358–0.972)	
Need of vasopressor support	1.878 (1.119–3.152)	2.042 (1.205–3.460)	2.158 (1.197–3.891)
Methylprednisolone < 1 mg/kg			0.500 (0.302–0.828)
Methylprednisolone > 1 mg/kg			0.479 (0.283–0.811)
*AUC (95% CI)*	0.712 (0.677; 0.746)	0.728 (0.694; 0.761)	0.746 (0.705; 0.787)
*Calibration, CITL (95% CI)*	0.001 (-0.306; 0.308)	0.0007 (-0.312;0.313)	< 0.001 (-0.398; 0.398)
*Slope (95% CI)*	1.025 (0.822; 1.228)	1.018 (0.827; 1.209)	1.000 (0.788; 1.214)

SAPS, Simplified Acute Physiology Score; NPPV: noninvasive positive pressure ventilation; N: L, neutrophil to lymphocyte ratio; AUC; area under the curve. OR, Odds Ratio; CI, confidence interval

The results of the mixed-effect analysis (Additional file 1: Table S9) showed that center had an effect associated with ICU mortality (MOR = 1.85). A sensitivity analysis of multiple imputation for ICU mortality showed that the estimates obtained from the imputed data and the estimates from the non-imputed data are almost identical (Additional file 1: Table S11).

## Discussion

This multicenter cohort study in 868 adult patients with severe COVID-19 undergoing invasive mechanical ventilation in Spain provides follow-up data concerning long-term survival after ICU admission. Independent predictors associated with 180-day mortality were older age, higher SAPS3, the need for norepinephrine, an increased neutrophil-to-lymphocyte ratio at ICU admission, a failed attempt of noninvasive positive pressure ventilation, and the lack of use of a selective digestive decontamination strategy during ICU stay. The proportion of patients who died varied widely between centers.

To the best of our knowledge, this is the first report dealing with and long-term outcomes in a large cohort of mechanically ventilated patients with severe COVID-19. Overall, in-hospital mortality was 40.5%, which is considerably lower than that in other high-income countries [18–24]. However, the age distribution and comorbidities of patients on mechanical ventilation was similar to that in these cited national studies. ICU mortality in our cohort was also similar (38%) to that in a large cohort of critically ill patients with COVID-19 in the Netherlands [24]. It is of note that patients in our study were followed for up to 180 days and survival rate was 59%.

Age has been one of the most controversial aspects during the outbreak of the COVID-19 pandemic*.* In our study, the unadjusted 180-day survival curves in severely ill patients over 70 years of age was above 50%, representing a better clinical outcome than in previous studies in older patients undergoing mechanical ventilation [26, 27]. Despite being considered clinically relevant, this difference in survival implies that age cannot be used as a clinical criterion to determine initiation of invasive mechanical ventilation. The availability of complementary clinical tools could aid decision-making with respect to initiating mechanical ventilation. In this sense, assessment of frailty has shown to be a robust guide to resource allocation in severely ill older patients with COVID-19 [28].

The use of non-invasive positive pressure ventilation (NPPV) for respiratory management of patients with severe acute respiratory distress is controversial [29]. Studies related to non-invasive positive pressure ventilation failure have shown that the delay in interrupting this ventilation may be associated with increased mortality [30–33]. Specifically, in a retrospective chart review study of patients with COVID-19 in Italy, mortality was 76% [34] in those who received noninvasive ventilation. In our study, the application of NPPV was not protocolized, and therefore, patients received any modality of NPPV (CPAP and BiPAP), and any kind of interfaces were used. Unfortunately, monitoring of respiratory parameters, modalities or management during sessions was not addressed due to the overwhelmed healthcare resources consumption during the pandemic, and use outside the ICU of these devices as well. Helmet noninvasive ventilation has been evaluated as an alternative for the noninvasive respiratory support of patients with hypoxemia with promising results in severe patients with COVID-19, but not found significant difference in the number of days free of respiratory support. Hence, monitoring of patients receiving noninvasive respiratory support during AHRF remains of paramount importance not to delay endotracheal intubation. On the other hand, probably patients that received NPPV as a prior attempt of noninvasive respiratory support were the most severe patients in which failed NPPV and, therefore, a selected type of patients. These could also be a reason for the worst outcomes.

The beneficial effect of the SDD strategy in mechanically ventilated critically ill patients has been widely studied, and meta-analysis based on individual patient data have consistently shown improved patient outcomes with this approach [35–37].

Indeed, the similarity in ICU mortality between our findings and Dutch study [24] may be explained, because SDD are currently widely used in the Spanish participating ICU and Dutch ICU [38]. This is the first study to report that SDD has the beneficial effect of decreasing ICU mortality in mechanically ventilated patients with severe COVID-19. Nevertheless, the low difference of the use of SDD between ICU survivors compared with ICU non survivors may limit to assume a direct impact in the outcomes. We did not, however, analyze the relationship between SDD-nosocomial infections in patients with severe COVID-19 and mortality, an objective for post-hoc analysis.

A hematological dysfunction defined as an increased neutrophil to absolute lymphocyte ratio (NLR) was recently described. The NLR has been identified as biomarker of higher in-hospital mortality [39]. Another study showed that NLR was significantly higher in hospitalized patients with severe forms of COVID-19 [40] and a higher NLR at hospital admission was associated with worse outcome [41, 42]. In our predictive model, an NLR higher than 15 on day 1 was significantly associated with increased mortality. Our data also confirm that NLR is a useful independent prognostic factor in COVID19 patients under mechanical ventilation.

We did not find that adjunctive therapies such as prone positioning or the use of neuromuscular blocking agents had a beneficial effect on mortality. It is important to highlight that the predictive model described here is based on the first 48 h of mechanical ventilation. One can, therefore, argue that these measures would not have been implemented for long enough to have a positive clinical impact on patients with severe COVID-19. Furthermore, this observational study collected the local clinical practice from centers during the pandemic outbreak. Most of the patients, therefore, received all these measures, thus limiting the possibility to extrapolate conclusions in the absence of adjusted and reliable comparisons.

According to recent evidence, treatment with systemic corticosteroids is associated with reduced mortality for critically ill patients with COVID-19 [43]. The clinical management of corticosteroids during the outbreak, nevertheless, was very heterogeneous. Indeed, in this recent meta-analysis, the effect of glucocorticoids on mortality at 28 days showed a marked heterogeneity (I^2^ 44%), suggesting that different populations or different associated treatment effects may play a role. On the other hand, the multivariate model for long-term outcomes (180-day mortality) showed that the administration of corticosteroids had a beneficial effect on mortality.

Finally, we have found that the need for low dosages of vasopressor support during ICU admission is a predictor for short-term mortality, but not for long term. Due to the observational design of the study we cannot verify the target for mean arterial pressure (MAP) or the hemodynamic management in these patients during ICU stay, neither if those patients had a component of *cardiac dysfunction* secondary to myocarditis that may influence in the prediction for unfavorable outcomes [44].

Our data indicate that 60% of all deaths among ventilated patients occurred in the first 30 days of ICU stay. These deaths were due to refractory hypoxemia and multiorgan failure, and were probably related to the development of progressive, fibrotic lung disease.

Although the virus is eradicated in the most severe COVID-19 patients, the cause of lung damage is not. Linked to the inflammatory response, lung fibrosis emerges as a secondary event related to the progression of the pathology and worse outcomes [45–48].

We acknowledge a number of limitations. First, due to the fact that most participating centers rapidly reached ICU saturation at the critical moment of the COVID-19 outbreak, and as intensivists were facing difficult decisions, not all patients admitted to the participating ICUs during the study period were collected. Nevertheless, we calculated a predefined sample size according to the protocol to reach significant power to detect clinical differences in outcomes. Second, it is plausible that differences in clinical outcomes may be explained by variability in clinical practice. Such an increased risk for ICU mortality can be explained by differences in practice between centers [49]. Finally, the predictive model could be affected by unmeasured confounders in patient populations in ICUs, explaining some of the variation observed in treatments and outcomes. Accordingly, our findings should be interpreted cautiously.

## Conclusions

Overall survival support in mecahnically ventilated patients with severe acute respiratory hypoxemic failure due to COVID-19 was slightly more than 50% at 180 days but this varied considerably between centers. The evolution and management of these patients during the ICU stay may influence long-term outcomes. Among the clinical modifiable factors that were predictors of death, our findings suggest that a failed attempt of NPPV may be associated with worse outcomes. However, the clinical challenge is still to early identify patients who benefit from NPPV and those who will have worse outcomes. Further studies based on personalized medicine are warranted to evaluate functional outcomes of this population and the multiple candidate therapies to detect treatment-responsive subgroups.

## Supplementary Information

**Additional file 1.** Additional figures and tables.

## Data Availability

The data sets generated and/or analyzed during the current study are publicly available due to ethics guidelines, but are available from the corresponding author on reasonable request.

## Abbreviations

Acute hypoxemic respiratory failure: AHRF
Acute respiratory distress syndrome: ARDS
Area under the receiver operating curve: AUROC
Calibration-in-the-large: CITL
Coronavirus disease 2019: COVID-19
Intensive care units: ICU
Median odds ratio: MOR
Multivariate imputation by chained equations: MICE
Noninvasive positive pressure ventilation: NPPV
Positive end-expiratory pressure: PEEP
Real-time reverse-transcriptase polymerase chain reaction: RT-PCR
Simplified Acute Physiology Score: SAPS
Standard deviation: SD
Selective digestive decontamination: SDD
