# Supplementary material for: Long-term survival of mechanically ventilated patients with severe COVID-19: an observational cohort study
Source: Ann Intensive Care. 2021 Oct 2;11:143. doi: 10.1186/s13613-021-00929-y (PMC8487336; doi:10.1186/s13613-021-00929-y)
Supplement: Supplementary file 1 — Additional file 1. Additional figures and tables. [file 13613_2021_929_MOESM1_ESM.docx]

**Additional material**

**Long-term survival of mechanically ventilated patients with severe COVID-19:** [**an observational cohort study**](https://erj.ersjournals.com/content/56/6/2003498)

Oscar Peñuelas P.h.D, Laura del Campo-Albendea MsC, Amanda Lesmes González de Aledo M.D., José Manuel Añón P.h.D, Carmen Rodríguez-Solís M.D., Jordi Mancebo P.h.D, Paula Vera M.D, Daniel Ballesteros P.h.D, Jorge Jiménez M.D., Emilio Maseda P.h.D, Juan Carlos Figueira P.h.D, Nieves Franco P.h.D , Ángela Algaba P.h.D , Juan Pablo Avilés M.D , Ricardo Díaz P.h.D , Beatriz Abad M.D, Alfonso Canabal P.h.D, Ana Abella P.h.D , Federico Gordo P.h.D, Javier García P.h.D, Jessica García-Suarez M.D, Jamil Cedeño M.D, Basilia Martínez-Palacios M.D, Eva Manteiga P.h.D, Óscar Martínez M.D, Rafael Blancas P.h.D, Tommaso Bardi M.D, David Pestaña P.h.D, José Ángel Lorente P.h.D, Alfonso Muriel P.h.D , Andrés Esteban P.h.D, Fernando Frutos-Vivar M.D.

This supplementary material has been provided by the authors to give readers additional information about their work.

**Index**

| List of collaborators | |
| --- | --- |
| Figure S1 | Flow chart of the study |
| Table S1 | Baseline characteristics of excluded patients |
| Table S2 | Characteristics of patients included in the analysis based on ICU mortality. |
| Table S3 | Serum biomarkers at admission in the intensive care unit |
| Table S4 | Ventilatory management and arterial blood gases within first week of mechanical ventilation |
| Figure S2 | Relationship between tidal volume (in ml/kg predicted body weight) and PEEP set within first 48 hours of mechanical ventilation |
| Table S5 | Comparison of early use (within first 48 hours) of adjuvant therapies to mechanical ventilation |
| Table S6 | Comparison in serum biomarkers between survivors and non-survivors |
| Table S7 | Complications in the first week of mechanical ventilation and other clinical outcomes |
| Figure S3 | Cumulative frequency distribution of ventilatory parameters within the first week of mechanical ventilation |
| Figure S4 | Kaplan-Meier survival curves at 180 after admission to ICU by subgroups |
| Table S8 | Univariate and multivariate analysis for ICU-mortality |
| Table S9 | Univariate and multivariate analysis for ICU-mortality |
| Table S10 | Random variability of mortality (Median Odds Ratio) |
| Figure S5 | Crude 180 day mortality and ICU mortality rates by centers |
|  | |
| Appendix S1: Operative definitions of variables. | |
| eReferences | |

**List of collaborators**

Hospital Universitario de Getafe

- Servicio de Medicina Intensiva: Inmaculada Alía, Jesús A. Álvarez, Enrique Calvo, Demetrio Carriedo, Karen Gómez, Gesly Gonçales, María González, Raquel Herrero, Carlos Jaramillo, Lucía López-Rodríguez, Diego Manzano, Enrique Mon, Kapil Nanwani, Lorena Oteiza, Anggie Rodríguez, Mercedes Rubio, Eva Tejerina, Dovami Vasco, Marina, Laura Whyte, Marina López.
- Servicio de Anestesia y Reanimación: Tomás Vega, Pablo Sayalero, Begoña Santaolalla, Enrique Roca, María Luz Pindado, Helena Pérez, Juan Pazos, Mario Morales, Adolfo Matilla, Sonia Martin Ventura, María Luisa Mariscal, Rafael López, Javier Jiménez, María Jesús Jiménez, María Teresa Gudin, María José G. Luna, Susana García González, Paloma Gallego, Elena Duro, Soledad Cortes, Rocío Castellanos, Patricia Carrasco, Mar Caro, Yolanda Cabrero, J. Ramón Cabañas, Antonio Bordas, Beatriz Alonso, Irene Alonso.
- Servicio de Cardiología: Paula Awamleh García.
- Servicio de Neurocirugía: Martin Tamarit, Yaiza López, Pedro Poveda, Juan Carlos Gómez-Angulo.
- Servicio de Angiología y Cirugía Vascular: Ignacio Michel, Cesar Varela, Alejandro Bravo, Carlos G. Cadenillas.
- Anatomía Patológica: Ana María Martín, Francisca Camacho, Joanny A. Duarte.
- Universidad Europea de Madrid: Anthony Silva, Santiago Ortiz.

Hospital Universitario Doce de Octubre: Isaías Martín, Francisco de Paula Delgado Moya, Reyes Muñoz Calahorro, Cristina Amírola Sarmiento de Sotomayor.

Hospital Universitario La Paz:

- Servicio de Medicina Intensiva: Andoni García-Muñoz, Cristina Arevalo, Claudio E. Gutiérrez.
- Servicio de Anestesia y Reanimación: Alejandro Suarez de La Rica, Itziar Insausti, Ana Montero Feijoo.

Hospital Santa Creu i Sant Pau: Jaume Baldirá Martínez de Irujo, Antoni Jordi Betbesé Roig, Marta Izura Gómez, Indalecio Morán Chorro, José Alberto Santos Rodríguez, Nuria Rodríguez Farré, Juan Carlos Suárez Montero, María Torrens Sonet, Lluís Zapata Fenor. Alejandra Cordón Abalde, Matías Nicolás Flores Orella, Carmen Gomila Sintes, Ana Segarra Martinez-Sahuquillo, Mikel Martos Mendizábal, Laura Mateo Marquina, Miguel Elías Morales Alarcón.

Hospital Universitario Puerta de Hierro Majadahonda:

- Servicio de Medicina Intensiva: Sandra Tejado Bravo, Diego Palacios Castañeda, Sara Alcántara Carmona, Carmen Arenas Mazarrota, Bárbara Balandín Moreno, Beatriz Castiñeiras Amor, Jorge Duerto Álvarez, Roberto Fernández Muñoz, Raquel Fernández Rivas, Inmaculada Fernández Simón, Rodrigo Fernández Tobar, Inés Lipperheide Valhonrat, Nuria Martínez Sanz, Alfonso Ortega López, Lucía Pérez Pérez, Marina Pérez Redondo, Manuel Pérez Torres, Miguel Valdivia de la Fuente, Héctor Villanueva Fernández, Juan José Rubio Muñoz.
- Servicio de Anestesia y Reanimación: Andrea Albajar, Nadia A. Abdallah, Maria Casado.

Hospital Universitario de Torrejón: Elena Abril, Elena González.

Hospital Universitario Gregorio Marañón: Alberto Garrido.

Hospital Universitario Severo Ochoa, Leganés-Madrid: Omar Fernando Fong Ruiz; Eva María Sánchez Sánchez; Irene Jiménez del Rio; Laura Asunción Matesanz Canencia; Mónica Fuentes Ponte; José Luis Flordelís La Sierra.

Hospital Universitario de Móstoles: Amparo del Caño García, Raquel Fernández García, Ana Ávarez Méndez, Leandro Castro Bournissen, Francy Carolina Dos Santos, Paula Pérez Fernández, Mercedes Martínez García, Maria Martín Ayuso, Diana Narvaez Cubillos.

Servicio de Medicina Intensiva. Hospital Universitario del Henares, Coslada, Madrid: Inés Torrejón Pérez; Leire López de la Oliva Calvo.

Hospital Universitario Del Tajo, Madrid: Blanca López Matamala.

Figure S1 – Flow chart of the study


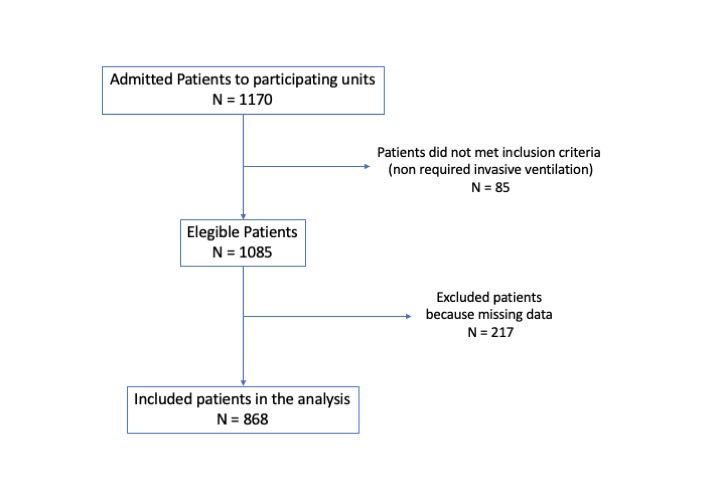


Table S1 – Baseline characteristics of excluded patients. Data are %, unless otherwise indicated.

|  | **Patients Excluded, N=217** |
| --- | --- |
| Age, years , mean (SD) | 58 (13) |
| Female | 28 |
| Comorbidities | |
| Hypertension | 43 |
| Obesity | 35 |
| Diabetes | 22 |
| Dyslipemia | 14 |
| Asthma | 6 |
| Ischemic heart disease | 6 |
| Chornic obstructive pulmonary disease | 9 |
| Chronic Kidney Disease | 4 |
| Immunodepression | 2 |
| Ictus | 2 |
| Autoimmune Disease | 2 |
| Hematological Malignancy | 2 |
| Previous therapy | |
| Angiotensin-converting enzyme inhibitors | 24.5 |
| Angiotensin II receptor blockers | 6 |
| Steroids | 3 |
| Days from initiation symptoms to admission at Hospital, median (P_25_, P_75_) | 7 (4, 9) |
| Management at hospitalization, | |
| Noninvasive positive pressure ventilation | 9 |
| Lopinavir/Ritonavir | 35 |
| Tocilizumab | 34 |
| Azithromycin | 51 |
| Steroids | 32 |
| Hydroxychloroquine | 68 |
| Baricitinib | 0.4 |
| Remdesivir | 2 |
| Anakinra | 1 |
| Arterial blood gases at ICU admission | |
| pH, mean (SD) | 7.33 (0.13) |
| PaCO_2_, mean (SD), mmHg | 49 (18) |
| Ratio PaO_2_/FiO_2_, mean (SD) | 118 (69) |

Table S2 – Clinical characteristics of patients included in the analysis based on ICU mortality. Data are n (%), unless otherwise indicated.

|  | **Overall**  **N = 868** | **Survivors**  **N = 533** | **Non-Survivors**  **N = 335** | **P value** |
| --- | --- | --- | --- | --- |
| Age. mean (SD), years | 64 [56; 71] | 59 (12) | 66 (10) | 0.000 |
| ≤40 | 48 (5) | 43 (8) | 5 (1) |  |
| 41-50 | 84 (10) | 62 (12) | 22 (7) |  |
| 51-60 | 199 (23) | 55 (29) | 44 (13) |  |
| 61-70 | 305 (35) | 183 (34) | 122 (36) |  |
| 71-80 | 222 (26) | 104 (19) | 118 (35) |  |
| >80 | 10 (1) | 4 (1) | 6 (2) |  |
| Sex, male | 625 (72) | 369 (69) | 256 (76) | 0.002 |
| Comorbidities |  | | |  |
| Hypertension | 401 (46) | 223 (42) | 178 (53) | 0.001 |
| Obesity | 268 (31) | 176 (33) | 92 (26) | 0.084 |
| Diabetes | 209 (24) | 103 (19) | 106 (32) | 0.000 |
| Cardiovascular disease | 428 (49) | 238 (45) | 190 (57) | 0.001 |
| Ischemic Cardiopathy | 65 (7) | 30 (6) | 35 (10) | 0.009 |
| Dyslipemia | 144 (17) | 103 (16) | 41 (17) | 0.006 |
| Pulmonary obstructive disease | 59 (7) | 24 (4) | 35 (10) | 0.001 |
| Asthma | 69 (8) | 49 (9) | 20 (6) | 0.087 |
| Cirrhosis | 5 (0.6) | 2 (0.4) | 3 (0.9) | 0.324 |
| Chronic Kidney Disease | 43 (4.9) | 11 (2.1) | 32 (9.6) | 0.000 |
| Immunodepression | 77 (8.9) | 33 (6.2) | 44 (13.1) | 0.005 |
| Ictus | 27 (3) | 13 (2) | 14 (4) | 0.151 |
| Autoimmune Disease | 26 (3) | 11 (2) | 15 (4) | 0.042 |
| Hematological Malignancy | 16 (2) | 7 (1) | 9 (3) | 0.143 |
| Symptoms |  |  |  |  |
| Fever | 679 (78) | 427 (80) | 252 (75) | 0.089 |
| Dyspnea | 640 (74) | 398 (75) | 242 (72) | 0.428 |
| Cough | 439 (51) | 282 (53) | 157 (47) | 0.08 |
| Asthenia | 182 (21) | 108 (20) | 74 (22) | 0.520 |
| Myalgias | 167 (19) | 119 (22) | 48 (14) | 0.004 |
| Gastrointestinal | 139 (16) | 84 (16) | 55 (16) | 0.797 |
| Headache | 57 (6.5) | 34 (6) | 23 (7) | 0.004 |
| Previous therapy |  | | |  |
| Statins | 288 (33.2) | 165 (30.9%) | 123 (36.7) | 0.079 |
| Angiotensin-converting enzyme inhibitors | 172 (19.8) | 95 (17.8) | 77 (23.0) | 0.063 |
| Angiotensin II receptor blockers | 122 (14.1) | 69 (12.9) | 53 (15.8) | 0.235 |
| Antiplatelets | 102 (11.8) | 49 (9.2) | 53 (15.8) | 0.003 |
| Beta-blockers | 97 (11.2) | 48 (9.0) | 49 (14.6) | 0.010 |
| Calcium Antagonists | 59 (6.8) | 38 (7.1) | 21 (6.3) | 0.624 |
| Anticoagulants | 48 (5.5) | 25 (4.7) | 23 (6.9) | 0.172 |
| Steroids | 34 (3.9) | 15 (2.8) | 19 (5.7) | 0.035 |
| Antiviral therapy |  |  |  |  |
| Lopinavir/Ritonavir | 577 (66) | 356 (67) | 221 (66) | 0.803 |
| Hydroxychloroquine | 716 (82) | 454 (85) | 262 (78) | 0.009 |
| Daily doses Hydroxychloroquine, mean (SD), mg | 423 (62) | 420 (55.5) | 430 (74) | 0.031 |
| Remdesivir | 12 (1) | 7 (1) | 5 (1) | 0.826 |
| Darunavir | 25 (3) | 15 (3) | 10 (3) | 0.884 |
| Immunomodulator therapy |  |  |  |  |
| Steroids | 419 (48) | 257 (48) | 162 (48) | 0.968 |
| Doses Steroids |  |  |  | 0.102 |
| Methylprednisolone < 1 mg/kg | 206 (24) | 137 (26) | 69 (21) |  |
| Methylprednisolone > 1 mg/kg | 213 (24) | 120 (22) | 93 (28) |  |
| Azithromycin | 328 (38) | 200 (37) | 128 (38) | 0.839 |
| Tocilizumab | 426 (49) | 135 (25) | 92 (27) | 0.486 |
| Interferon | 129 (15) | 74 (14) | 55 (16) |  |
| Noninvasive respiratory support at ICU before intubation |  | | | 0.004 |
| Oxygen mask alone | 772 (88.9) | 479 (89.9) | 293 (87.5) |  |
| High flow oxygen nasal cannula | 6 (0.7) | 5 (0.9) | 1 (0.3) |  |
| Non-invasive positive pressure ventilation | 77 (8.9) | 43 (8.6) | 39 (11.6) |  |
| Non-invasive positive pressure ventilation and high flow oxygen nasal cannula | 13 (1.5) | 6 (1.1) | 7 (2.1) |  |
| PCR negative in patients with first PCR positive | 305 (46.9) | 237 (57.8) | 48 (20.0) | 0.127 |
| Days from first PCR to negative PCR | 23 (14,31) | 23 (15, 31) | 21 (13, 28) | 0.039 |

Table S3 – Serum biomarkers at admission in the intensive care unit.

|  | **Patients with data** | **Overall** | **Survivors** | **Non survivors** | **p-values** |
| --- | --- | --- | --- | --- | --- |
| Creatinine, median (P_25_, P_75_), mg/dl | 857 | 0.8 (0.6, 1.1) | 0.8 (0.6, 1.0) | 0.9 (0.7, 1.3) | 0.077 |
| Urea, median (P_25_, P_75_), mg/dl | 769 | 45 (31, 62) | 41 (30, 57) | 45 (32, 62) | 0.030 |
| LDH, median (P_25_, P_75_), UI/L | 738 | 538  (413, 723) | 510 (406, 677) | 584 (433, 769) | 0.189 |
| Bilirubin, median (P_25_, P_75_), mg/dl | 728 | 0.6 (0.4; 1.0) | 0.6 (0.4; 1.0) | 0.6 (0.4; 1.0) | 0.332 |
| ASAT, median (P_25_, P_75_), UI/L | 747 | 50 (35, 73) | 50 (34, 72.5) | 49.5 (38, 72.5) | 0.370 |
| ALAT, median (P_25_, P_75_), UI/L | 782 | 40 (26, 65) | 40.5 (26.5, 62) | 36 (25, 67) | 0.151 |
| Creatinkinase, median (P_25_, P_75_), mg/dl | 412 | 106 (55, 249) | 101.5 (54, 239) | 113.5 (59, 253.5) | 0.078 |
| Lactate, median (P_25_, P_75_), mmol/l | 758 | 1.4 (1.1, 2.0) | 1.6 (1.2, 2.3) | 1.3 (1.0, 1.8) | 0.058 |
| Troponin, median (P_25_, P_75_), ng/ml | 291 | 16 (9, 38) | 15 (9, 33) | 17 (11, 50) | 0.413 |
| Neutrophil, median (P_25_, P_75_), cells/ µl | 850 | 7800  (5010, 11700) | 7490 (5000, 10700) | 8385 (5090, 13800) | 0.002 |
| Lymphocyte, median (P_25_, P_75_), cells/ µl | 850 | 620 (410, 900) | 650 (440, 950) | 600 (390, 800) | 0.011 |
| Ratio Neutrophil: Lymphocyte, median (P_25_, P_75_) | 850 | 13 (7, 22) | 11 (7, 18) | 16 (9, 28) | 0.428 |
| Platelets, median (P_25_, P_75_), cells x10^3^/ µl | 850 | 238 (177, 316) | 252 (187, 324) | 220 (163, 301) | 0.113 |
| Prothrombin Activity, median (P_25_, P_75_), % | 636 | 78 (67, 92) | 79 (68, 92) | 76 (65, 91) | 0.207 |
| D-Dimer, median (P_25_, P_75_), µg/ml | 585 | 3.5 (1, 17) | 4 (1, 20) | 3.5 (1, 12) | 0.425 |
| C-Reactive Protein, median (P_25_, P_75_), mg/L | 772 | 116 (24, 237) | 119 (24,238) | 105 (25, 240) | 0.324 |
| Procalcitonin, median (P_25_, P_75_), ng/ml | 458 | 0.27  (0.13, 0.70) | 0.27 (0.12, 0.71) | 0.27 (0.15, 0.67) | 0.193 |
| Interleukin-6, median (P_25_, P_75_), pg/ml | 162 | 230 (66, 694) | 199 (57, 564) | 278 (80, 900) | 0.078 |

Table S4 - Ventilatory management and arterial blood gases within first week of mechanical ventilation. Data are n (%), unless otherwise indicated.

|  | **Overall**  **N = 868** | **Survivors**  **N = 533** | **Non-Survivors**  **N = 335** | **p-value** |
| --- | --- | --- | --- | --- |
| Highest Tidal volume, mean (SD), ml | 488.3 (74.2) | 483.9 (72.8) | 494.8 (75.8) | 0.022 |
| ml/kgPBW | 7.8 (1.5) | 7.7 (1.4) | 7.9 (1.7) | 0.059 |
| Highest PEEP, mean (SD), cm of water | 13.7 (2.8) | 13.7 (2.7) | 13.8 (2.9) | 0.318 |
| Highest plateau pressure, mean (SD), cm of water# | 27.9 (4.6) | 27.5 (4.5) | 28.5 (4.7) | 0.004 |
| Highest driving pressure, mean (SD), cm of water# | 15.7 (4.4) | 15.3 (4.1) | 16.3 (4.7) | 0.003 |
| Worst pH, mean (SD) | 7.3 (0.09) | 7.3 (0.09) | 7.2 (0.10) | < 0.001 |
| Worst PaCO_2_ , mean (SD) | 38.5 (7.4) | 37.9 (6.4) | 39.6 (8.7) | < 0.001 |
| Worst ratio PaO_2_ to FiO_2,_ mean (SD), mmHg | 94.5 (36.3) | 99.4 (38.1) | 86.6 (31.7) | < 0.001 |
| Highest ventilatory ratio, mean (SD) | 2.6 (0.9) | 2.5 (0.8) | 2.9 (1.0) | < 0.001 |
| Adjuvant supportive therapies |  |  |  |  |
| ECMO | 27 (3) | 18 (3) | 9 (3) | 0.568 |
| Inhaled Nitric Oxide | 29 (3) | 13 (2) | 16 (5) | 0.062 |
| Prone position | 573 (66) | 347 (65) | 226 (68) | 0.475 |
| Neuromuscular blockers | 704 (81) | 429 (80) | 275 (82) | 0.557 |

* Registered in 387 patients

# Registered in 609 patients

Figure S2 - Relationship between tidal volume (in ml/kg predicted body weight) and PEEP set within first 48 hours of mechanical ventilation in survivors and non-survivors.Table S5 - Comparison of early use (within first 48 hours) of adjuvant therapies to mechanical ventilation with ICU mortality.

|  | **Survivors**  N = 533 | **Non-survivors**  N = 335 | **Univariate Odds Ratio**  **(95% confidence interval)** |
| --- | --- | --- | --- |
| Prone position | 291 (55) | 176 (52) | 0.920 (0.699 – 1.210) |
| ECMO | 5 (1) | 6 (2) | 1.925 (0.583 – 6.360) |
| NO inhaled | 5 (1) | 7 (2) | 2.253 (0.709 – 7.159) |
| Steroids | 257 (48) | 162 (48) | 1.005 (0.765 – 1.321) |
| Neuromuscular blockers | 396 (74) | 236 (70) | 0.824 (0.608 – 1.118) |

Table S6. Comparison in serum biomarkers between survivors and non-survivors within the first week from beginning of mechanical ventilation.

|  | **Patients**  **with data** | **Survivors** | **Non-Survivors** | **P value** |
| --- | --- | --- | --- | --- |
| Creatinine, median (P_25_, P_75_), mg/dl | 867 | 0.9 (0.7, 1.4) | 1.3 (0.9, 2.3) | <0.001 |
| LDH, median (P_25_, P_75_), UI/L | 834 | 545  (438, 706) | 632  (489, 817) | <0.001 |
| Bilirrubin, median (P_25_, P_75_), mg/dl | 848 | 0.9 (0.6, 1.5) | 0.9 (0.6, 1.8) | 0.402 |
| Lactate, median (P_25_, P_75_), mmol/l | 863 | 2.2 (1.7, 2.8) | 2.4 (1.9, 3.2) | 0.002 |
| Troponin, median (P_25_, P_75_), ng/ml | 440 | 20 (10, 68) | 22 (12, 68) | 0.629 |
| Neutrophil, median (P_25_, P_75_), cells/μl | 863 | 7500  (5022, 11047) | 8290  (4995, 13720) | 0.043 |
| Lymphocyte, median (P_25_, P_75_), cells/μl | 863 | 430  (300, 607) | 340  (200, 510) | <0.001 |
| Ratio Neutrophil: Lymphocyte, median (P_25_, P_75_) | 863 | 20 (13,32) | 30 (17, 51) | <0.001 |
| Platelets, median (P_25_, P_75_), cells x10^3^/μl | 866 | 357  (269, 448) | 291  (209, 363) | <0.001 |
| D-Dimer, median (P_25_, P_75_), μg/ml | 783 | 4 (1.2, 12) | 6 (2**·**.5, 20) | 0.730 |
| Fibrinogen, median (P_25_, P_75_), mg/dl | 671 | 585  (500, 806) | 575  (416,795) | 0.746 |
| Procalcitonin, median (P_25_, P_75_), ng/ml | 672 | 0.4 (0.1, 0.9) | 0.5 (0.2, 1.9) | 0.007 |
| Interleukin-6, median (P_25_, P_75_), pg/ml | 238 | 227  (62, 646) | 301 (80,900) | 0.132 |

Table S7 – Complications within the first week of mechanical ventilation, during global ICU stay, and other clinical outcomes. Data are n (%), unless otherwise indicated.

|  | **Overall**  N = 868 |
| --- | --- |
| Acute respiratory distress syndrome | 700 (81) |
| Barotrauma | 49 (6) |
| Pneumothorax | 23 (3) |
| Tube obstruction | 94 (11) |
| Ventilator associated pneumonia | 206 (24) |
| Empyema | 79 (9) |
| Bacteremia | 241 (28) |
| Myocarditis | 24 (3) |
| Cardiovascular dysfunction | 557 (64) |
| Pulmonary Embolism | 52 (6) |
| Renal dysfunction | 114 (13) |
| Renal Replacement Therapy | 77/114 (67) |
| **Clinical outcomes** | |
| Scheduled extubation | 526 (61) |
| Reintubation | 71 (13) |
| Tracheotomy | 324 (37) |
| Percutaneous | 176 (54) |
| Surgical | 148 (46) |
| Time to tracheotomy, days, median, (P_25_, P_75_) | 15 [11; 20] |
| Duration of mechanical ventilation, days, median, (P_25_, P_75_) | 11 [7; 21] |
| Withdrawal of life support | 163 (18.8) |
| ICU mortality | 334 (38.5) |
| 28-day mortality | 184 (21.2) |
| Hospital mortality | 352 (40.5) |
| Length of ICU stay, days, median (P_25_, P_75_) | 16 [10; 30] |
| Length of hospital stay, median (P_25_, P_75_) | 34 [20; 70] |
| ICU readmission | 28 (3.2) |
| **Incidence of complications during ICU stay** | |
| Invasive broncopulmonary aspergillosis | 11 (1.2) |
| Viral pneumonia (herpes virus family or CMV) | 34 (3.9) |
| Myocarditis | 42 (4.8) |
| Cardiovascular dysfunction | 248 (28.5) |
| Deep venous thrombosis | 46 (5.2) |
| Acute pulmonary embolism | 84 (9.6) |
| ICU-acquired paresis | 283 (32.6) |
| Delirium | 262 (30.1) |
| Severe event related with therapeutic anticoagulation | 34 (3.9) |
| Encephalitis | 10 (1.1) |
| **Reason of death during ICU admission, (%)** | |
| Refractory hypoxemia | 31% |
| Refractory shock | 23% |
| Septic shock | 12% |
| Others | 14% |
| Acute pulmonary thromboembolism | 6% |
| Others | 7.5% |

Figure S3. Cumulative frequency distribution of ventilatory parameters within the first week of mechanical ventilation: A. Volume tidal; B) Driving pressure; C) Ventilatory ratio.


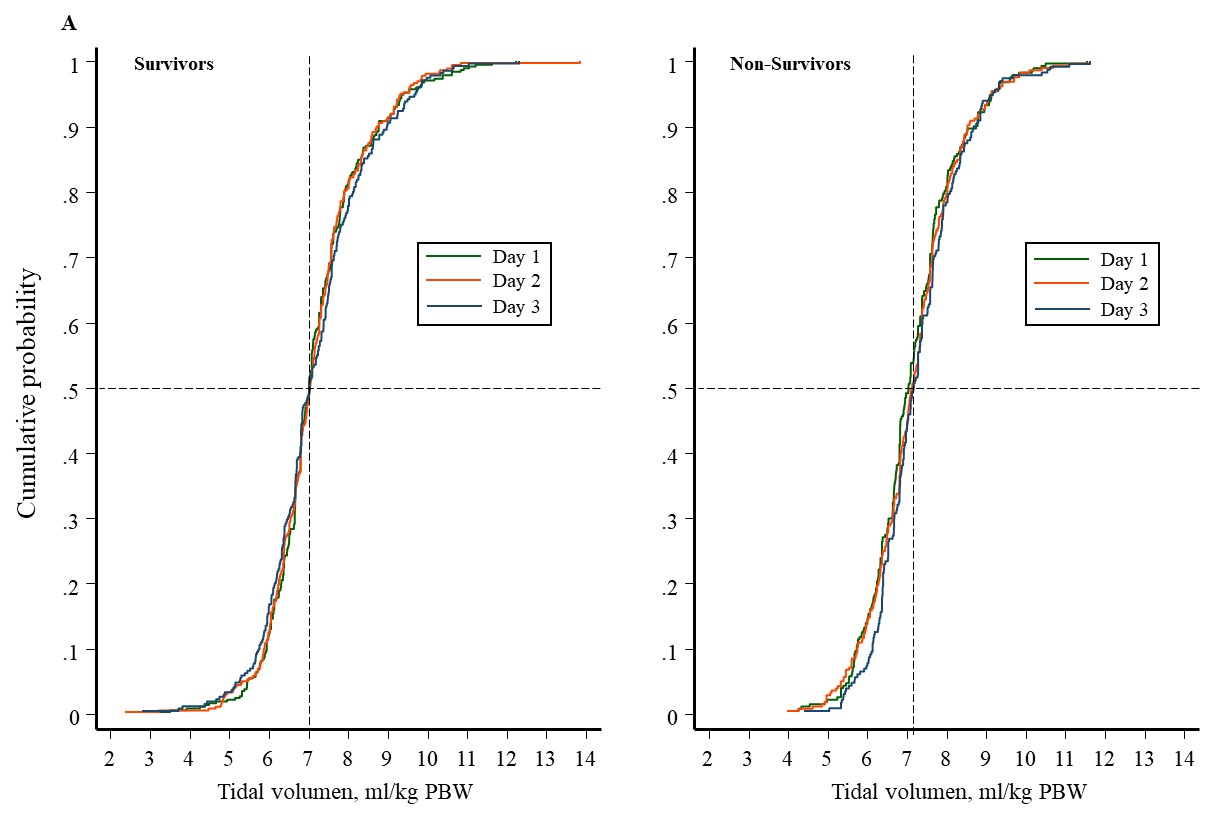


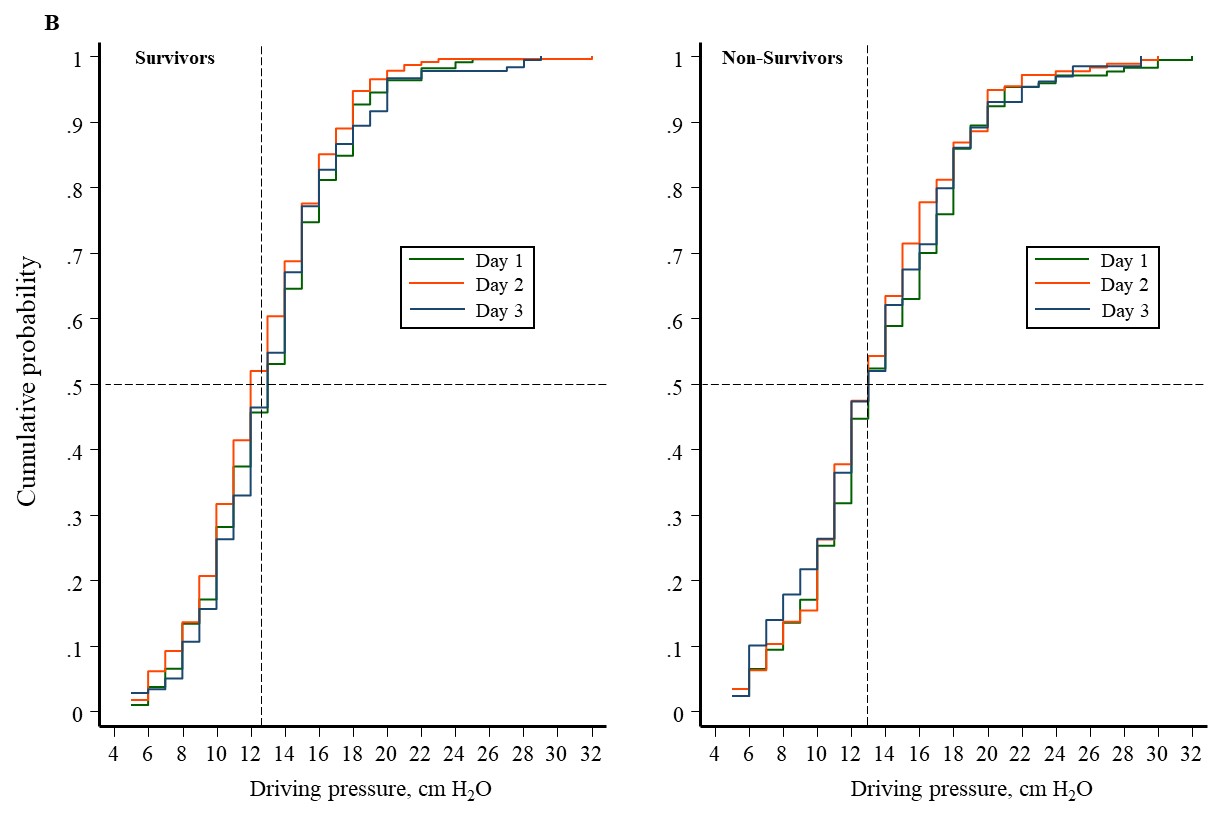


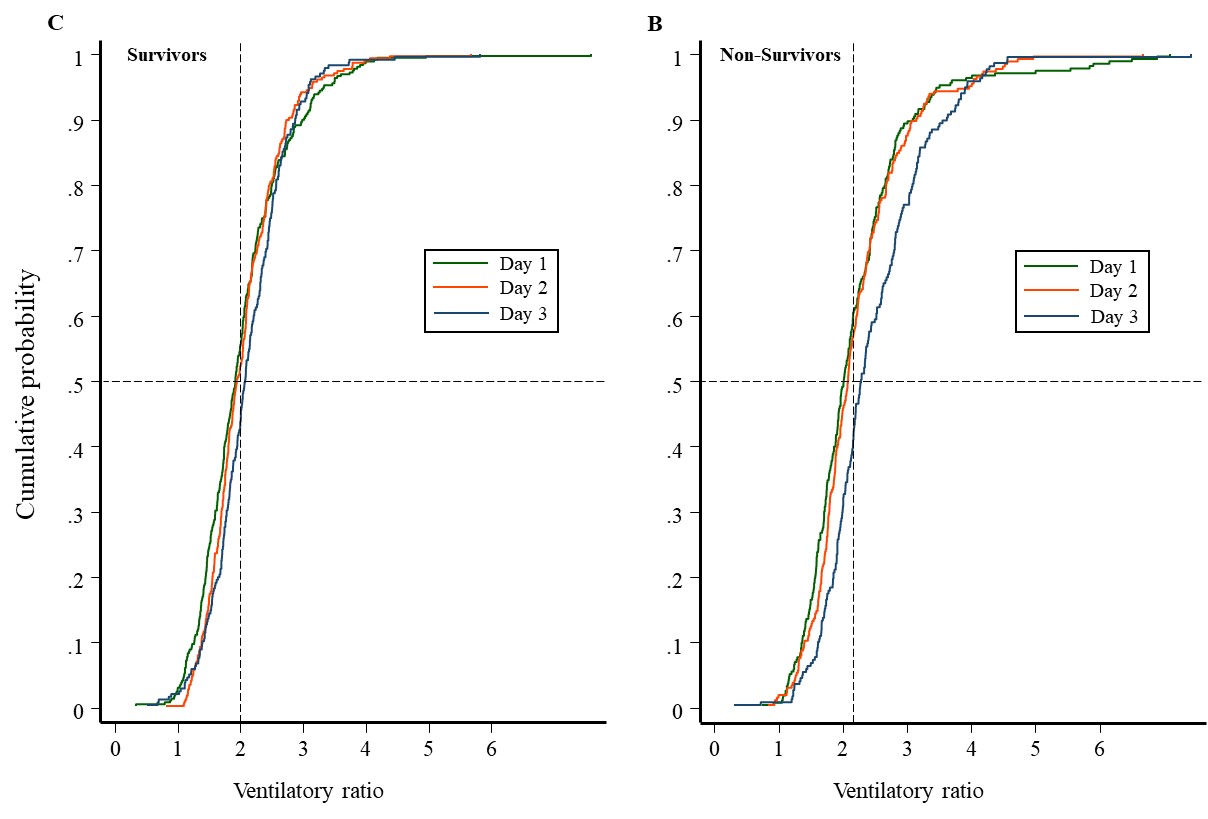


Figure S4. Kaplan-Meier survival curves at 180 after IUC admission.

- By Age: 180-day survival

*
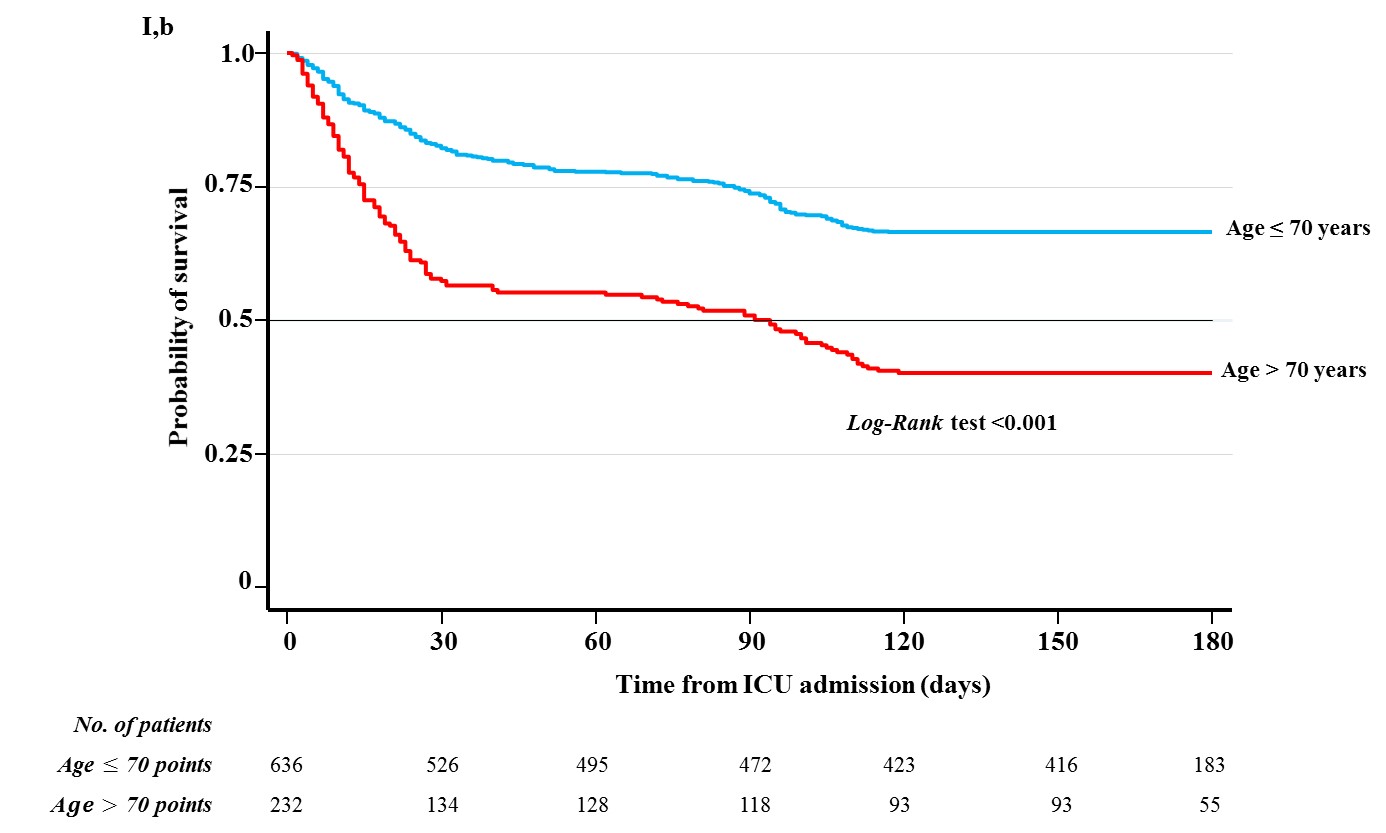
*

- By SAPS3 at ICU admission. 180-day survival

*
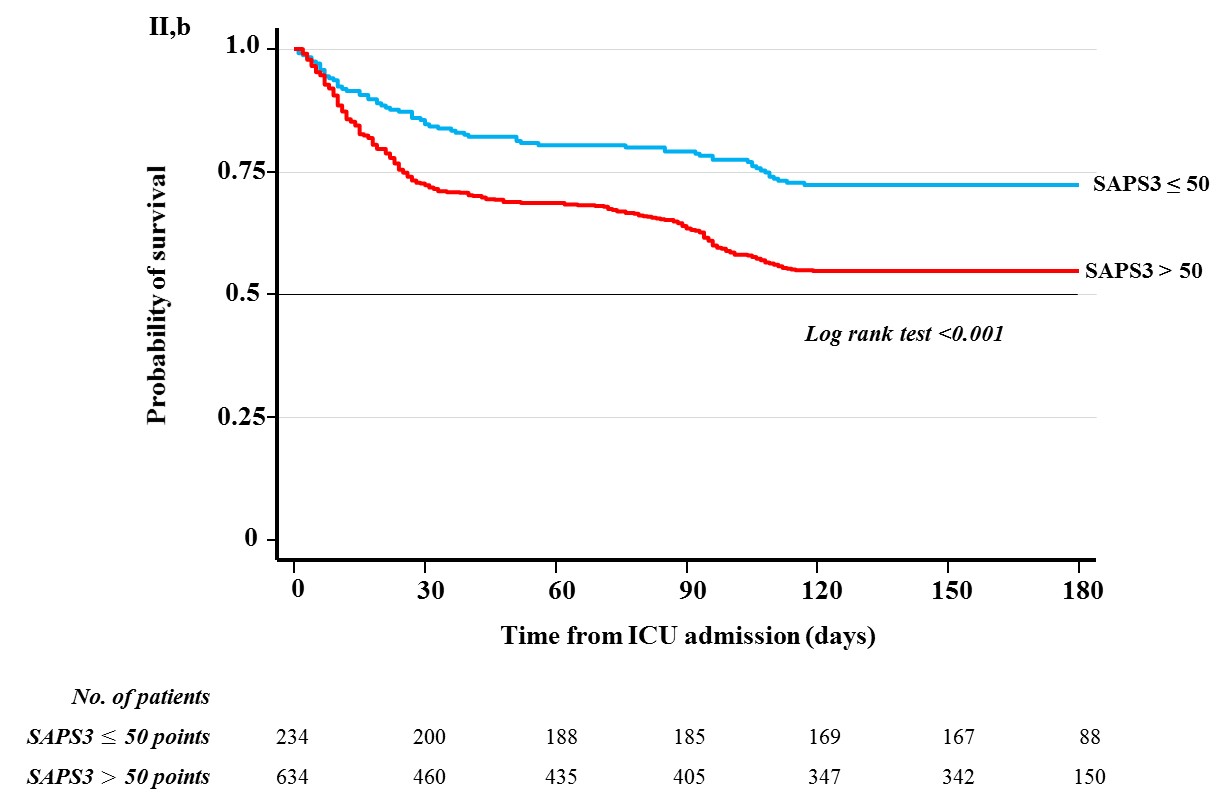
*

- By Berlin ARDS criteria: 180-day survival


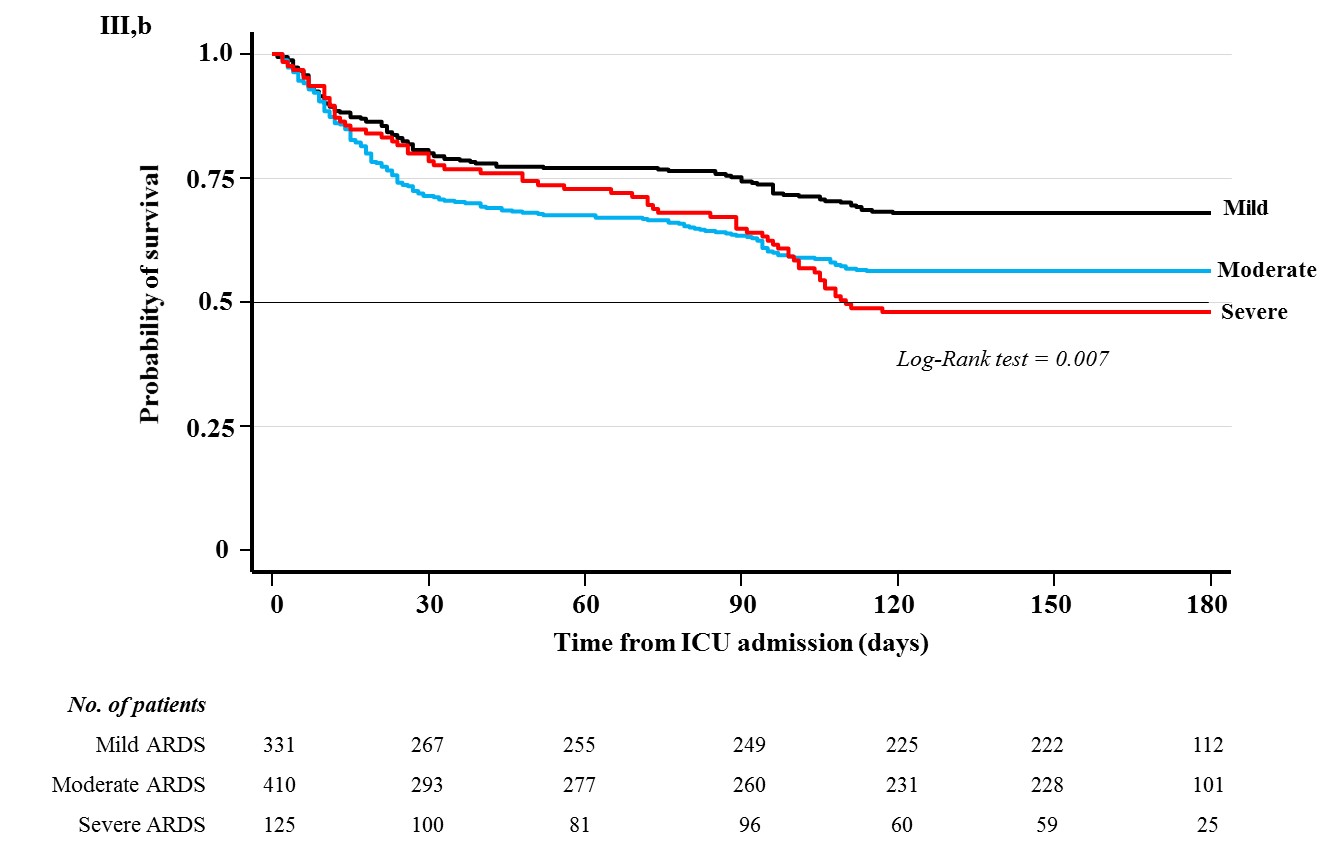


- By noninvasive ventilatory support previous to admission in the ICU) 180-day su
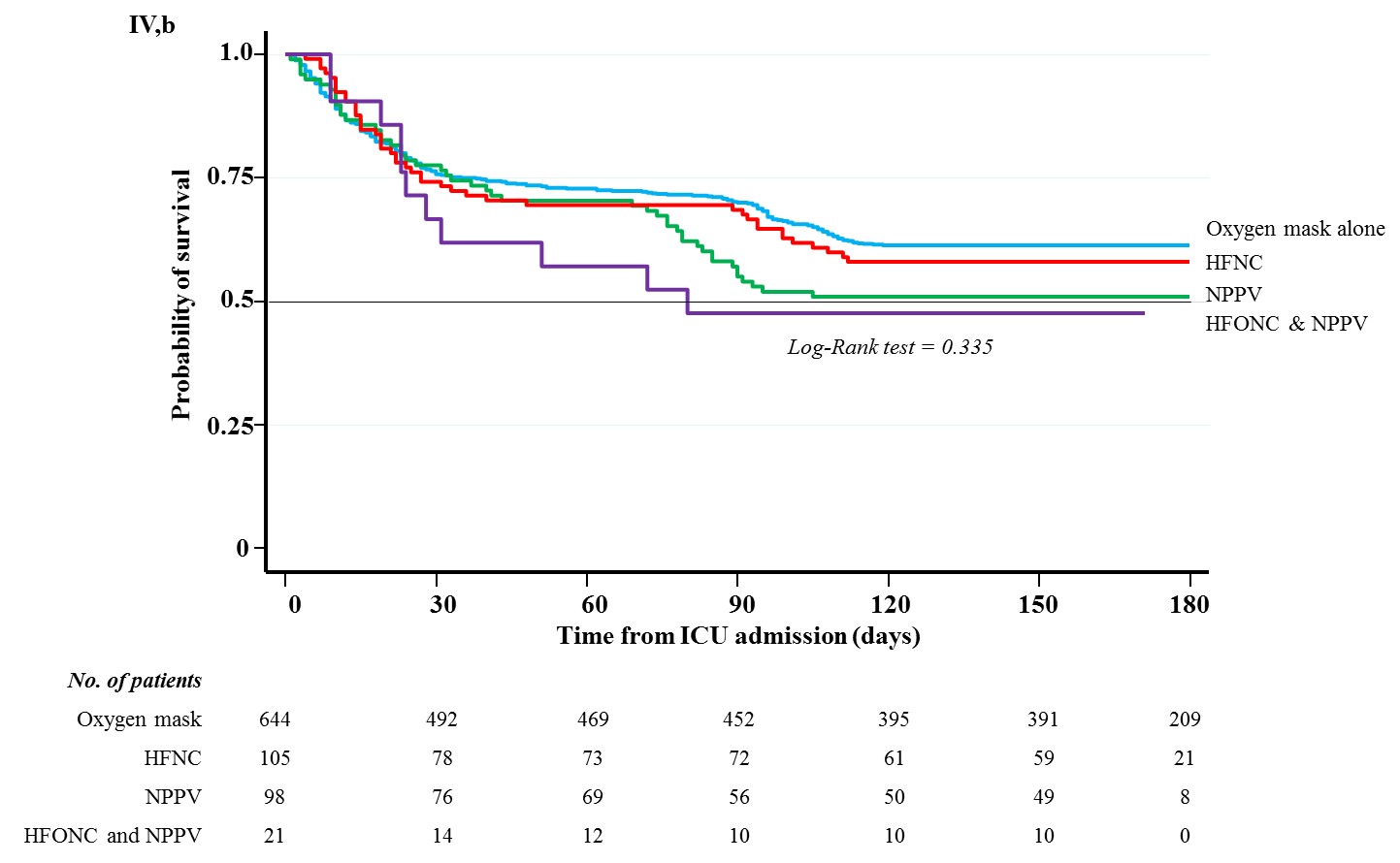
rvival

Table S8- Univariate and multivariate analysis for ICU-mortality. Data are n (%), unless otherwise indicated.

|  | **Survivors**  N=533 | **Non-Survivors**  N=335 | **Univariable OR (CI 95%)** | **P-value** | **Multivariable OR (CI 95%)** | **P-value** |
| --- | --- | --- | --- | --- | --- | --- |
| Age, mean (SD), years | 59 (12) | 66 (10) | 1.061  (1.046 – 1.077) | <0.001 | 1.049  (1.032-1.066) | <0.001 |
| Sex, male | 369 (69.2) | 256 (76.4) | 1.440  (1.054 – 1.968) | 0.022 |  |  |
| Comorbidities |  | | | | | |
| Hypertension | 223 (41.8) | 178 (53.1) | 1.576  (1.197 - 2.075) | 0.001 |  |  |
| Obesity | 176 (33.3) | 92 (26.0) | 0.7679  (0.569 - 1.037) | 0.085 |  |  |
| Diabetes | 103 (19.3) | 106 (31.6) | 1.932  (1.410 - 2.648) | <0.001 |  |  |
| Inpatient Medication |  | | | | | |
| Angiotensin-converting enzyme inhibitors | 95 (17.8) | 77 (23.0) | 1.376  (0.982 - 1.929) | 0.064 |  |  |
| Angiotensin II receptor blockers | 69 (12.9) | 53 (15.8) | 1.264  (0.858 - 1.862) | 0.236 |  |  |
| Steroids | 15 (2.8) | 19 (5.7) | 2.076  (1.040 - 4.145) | 0.038 |  |  |
| Non invasive Respiratory support at ward |  | | | | | |
| Oxygen mask alone | 408 (76.6) | 226 (70.5) | *[reference]* |  | *[reference]* |  |
| High flow oxygen nasal cannula | 65 (11.9) | 40 (12.1) | 1.064  (0.695 - 1.628) | 0.775 | 1.033  (0.611 - 1.745) | 0.904 |
| Non-invasive positive pressure ventilation | 50 (9.4) | 48 (14.3) | 1.660  (1.083 - 2.544) | 0.020 | 2.132  (1.280 - 3.551) | 0.004 |
| Non-invasive positive pressure ventilation and High flow oxygen nasal cannula | 10 (1.9) | 11 (3.3) | 1.901  (0.796 - 4.545) | 0.148 | 2.398  (0.899 - 6.400) | 0.081 |
| SAPS3, median (P_25_, P_75_), points | 54  [48.5, 61] | 58.5  [52; 68] | 1.044  (1.03 – 1.06) | <0.001 | 1.025  (1.008-0.041) | 0.002 |
| Days until intubation, median (P_25_, P_75_) | 3 [1; 5] | 3 [1; 7] | 1.050  (1.021 - 1.079) | 0.001 |  |  |
| Ventilatory management |  | | | | | |
| Tidal volume, mean (SD), ml/kg PBW | 7.2 (1.2) | 7.2 (1.6) | 1.010  (0.898 - 1.136) | 0.862 |  |  |
| PEEP, mean (SD, cm of water | 13 (3) | 13 (3) | 0.984  (0.936 - 1.035) | 0.543 |  |  |
| Continuous infusion of neuromuscular blockers | 396 (74.3) | 236 (70.5) | 0.825  (0.608 - 1.118) | 0.215 |  |  |
| Prone position | 291 (46.6) | 176 (52.5) | 0.921  (0.699 - 1.211) | 0.554 |  |  |
| Arterial blood gases |  | | | | | |
| Ratio PaO_2_/FiO_2_, mean (SD) | 108 (47) | 100 (48) | 0.997  (0.994 -0.999) | 0.033 |  |  |
| Ventilatory ratio, mean (SD) | 2.0 (0.8) | 2.2 (0.9) | 1.376  (1.138 - 1.665) | 0.001 |  |  |
| Serum biomarkers |  |  |  |  |  |  |
| Ratio Neutrophil/Lymphocyte, median (P_25_, P_75_) | 15  [9; 26] | 22  [12; 37] | 1.018  (1.001 – 1.026) | <0.001 | 1.009  (1.002-1.016) | 0.013 |
| D-Dimer, median (P_25_, P_75_), µg/ml, | 2.9  [0.9; 12.3] | 3.9  [1.4; 14.7] | 1.001  (0.94 - 1.008) | 0.702 |  |  |
| PCR, median (P_25_, P_75_), mg/L | 134  [30; 250] | 153  [31; 253] | 1.001  (0.999 - 1.002) | 0.132 |  |  |
| Drug therapy |  | | | | | |
| Selective Digestive Decontamination | 301 (56.5) | 149 (44.5) | 0.617  (0.469 - 0.813) | 0.001 | 0.587  (0.358 - 0.963) | 0.035 |
| Immunomodulator therapy | 210 (39.4) | 137 (40.9) | 1.064  (0.805 - 1.406) | 0.661 |  |  |
| Steroids |  | | | | | |
| No | 276 (51.8) | 173 (51.6) | *[reference]* |  |  |  |
| Methylprednisolone ≤ 1 mg/kg | 137 (25.7) | 69 (20.6) | 0.803  (0.568 - 1.136) | 0.215 |  |  |
| Methylprednisolone > 1 mg/kg | 120 (22.5) | 93 (27.8) | 1.236  (0.888 - 1.721) | 0.209 |  |  |
| Antiviral therapy | 492 (92.3) | 304 (90.8) | 0.817  (0.502 - 1.331) | 0.417 |  |  |
| Therapeutic anticoagulation therapy | 25 (4.7) | 23 (6.9) | 0.936  (0.806 - 1.088) | 0.388 |  |  |
| Norepinephrine^a^ |  | | | | | |
| No | 203 (38.1) | 103 (30.8) | *[reference]* |  | *[reference]* |  |
| Low doses | 44 (8.3) | 61 (18.2) | 2.732  (1.735 - 4.304) | <0.001 | 1.879  (1.119 - 3.153) | 0.017 |
| High doses | 286 (53.7) | 171 (51.0) | 1.178  (0.869 - 1.596) | 0.289 | 0.968  (0.685 - 1.369) | 0.855 |

a.- Doses for norepinephrine were considered according to the cardiovascular Sepsis-related Organ Failure Assessment (SOFA score) as: low doses defined as SOFA ≤ 3 points (≤ 0.1 μg/kg/min); high doses defined as SOFA > 3 points (> 0.1 μg/kg/min). Abbreviations: PEEP, positive end-expiratory pressure; OR, odds ratio; CI confidence interval.

Table S10. - Random variability of mortality (Median Odds Ratio). Median odds ratio (MOR) is defined as the median value of the odds ratio between the ICU at highest risk of mortality and the ICU at lowest risk when randomly picking out two units. The MOR can be conceptualized as the median increased risk of mortality that a patient would have if he or she moved to an ICU with a higher risk.

|  | **ICU**  **Mortality** | **Hospital**  **Mortality** | **28-day**  **Mortality** | **180-day**  **Mortality** |
| --- | --- | --- | --- | --- |
| **Overall effect**  (including all ICU) | 1.85 | 1.87 | 2.27 | 1.93 |
| **ICUs with ≤ 50 patients** | 1.75 | 1.77 | 2.11 | 1.93 |
| **ICUs with > 50 patients** | 1.66 | 1.72 | 2.07 | 1.79 |

Table S11. – Sensitivity analysis of multiple imputation for ICU mortality.

|  | **Multivariate OR (CI 95%)**  Imputed data | **P-value** | **Multivariate OR (CI 95%)**  Non-imputed data | **P-value** |
| --- | --- | --- | --- | --- |
| Age, years | 1.049  (1.032-1.066) | <0.001 | 1.06  (1.040 – 1.081) | <0.001 |
| Respiratory support at ward |  | | | |
| Oxygen mask alone | *[reference]* |  | *[reference]* |  |
| High flow oxygen nasal cannula | 1.033  (0.611 - 1.745) | 0.904 | 1.044  (0.566 – 1.927) | 0.888 |
| Non-invasive positive pressure ventilation | 2.132  (1.280 - 3.551) | 0.004 | 1.709  (0.910 – 3.212) | 0.095 |
| Non-invasive positive pressure ventilation and high flow oxygen nasal cannula | 2.398  (0.899 - 6.400) | 0.081 | 3.525  (0.911 – 13.636) | 0.068 |
| SAPS3, points | 1.025  (1.008-0.041) | 0.002 | 1.021 | 0.019 |
| Neutrophil to Lymphocyte Ratio | 1.009  (1.002-1.016) | 0.013 | 1.016 | 0.004 |
| Selective Digestive Decontamination | 0.587  (0.358 - 0.963) | 0.035 | 0.527  (0.295 – 0.941) | 0.030 |
| Norepinephrine^a^ |  | | | |
| No | *[reference]* |  | *[reference]* |  |
| Low doses | 1.879  (1.119 - 3.153) | 0.017 | 2.989  (1.612 – 5.473) | <0.001 |
| High doses | 0.968  (0.685 - 1.369) | 0.855 | 1.054 | 0.792 |

Abbreviations: SAPS, Simplified Acute Physiology Score 3; OR, odds ratio; CI confidence interval. a.- Doses for norepinephrine were considered according to the cardiovascular Sepsis-related Organ Failure Assessment (SOFA score) as: low doses defined as SOFA ≤ 3 points (≤ 0.1 μg/kg/min); high doses defined as SOFA > 3 points (> 0.1 μg/kg/min).

Figure S5. Crude 180-day mortality and ICU mortality rates for each participating center. Black upper and lower lines represents 95% confidence interval. A) ICU mortality; b) 180 day mortality.

Appendix S1. Operative definitions of variables included in the protocol of the study

| METHODS |  |
| --- | --- |
| Relevant variables | Those variables includes in the multivariate analysis (age, sex, SAPS3, use of corticosteroids, neutrophils to lymphocytes ratio, use of noninvasive respiratory devices, vasopressor support, use of selective digestive decontamination [SDD]), or variables related with clinical outcomes (ICU mortality, hospital mortality, date of ICU discharge, date hospital discharge, duration ventilator support, date of endotracheal intubation, date extubation) were all pre-defined as relevant variables and implied the categorization of patients as missing. |
| COMORBILITIES |  |
| Cardiovascular disease | Includes: ischemic cardiopathy, ictus, atrial fibrillation. |
| Metastatic tumor | Any solid organ tumor with stage M1 of the TNM classification. |
| Hematologic cancer: | Includes lymphomas, leukemia, multiple myeloma, myelodysplastic and myeloproliferative syndromes. |
| Immunosuppression | defined as neutropenia below 1000 / mm3 after bone marrow transplantation or chemotherapy, immunosuppressive therapy for solid organ transplantation, or any auto-immune disease requiring corticosteroid therapy of at least 20 mg / day for at least 3 weeks |
| PREVIOUS CHRONIC TREATMENTS | It refers to chronic treatments, with a duration of at least 6 previous months. The name of the active or commercial principle is not required |
| Use of Selective Digestive Decontamination (SDD) strategy | SDD uses non-absorbable oral and enteric antibiotics (polymyxin E, tobramycin and nistatine [PTN]), and a short course (4 days) of parenteral antibiotics (IV cefotaxime): * if methicillin-resistant *S. aureus* (MRSA) endemic (>1 case/month with a diagnostic sample positive for MRSA over a 6 month perioid): add oropharyngeal gel and/or intestinal solution of vancomycin.   - High level of hygiene to prevent exogenous infections. - Also topical PTN on the tracheostomy in tracheostomized patients to control exogenous lower airway infections. - Surveillance cultures of throat and rectum — on admission and twice weekly — monitor the effectiveness and to detect resistance. |
| RESPIRATORY COMPLICATIONS |  |
| Acute respiratory distress syndrome (ARDS) | Based on the Berlin definition: development of respiratory failure in the first week from a known clinical insult (trauma, pancreatitis, massive transfusion, pneumonia, gastric aspiration), not explained by heart failure or fluid overload; PaO_2_ FiO_2_ ratio <200 with PEEP ≥ 5 cmH_2_O and bilateral infiltrates on the chest radiograph (1). |
| Barotrauma | Appearance of air leak (pneumothorax, subcutaneous emphysema, pneumomediastinum, pneumopericardium) secondary to invasive mechanical ventilation |
| Iatrogenic pneumothorax | Appearance of pneumothorax after a central venous catheter (jugular, subclavian vein) cannulation procedure in the 72 hours prior to the event |
| Endotracheal tube obstruction | Need to change the orotracheal tube due to obstruction secondary to secretions |
| CARDIOVASCULAR COMPLICATIONS |  |
| Acute Thrombotic events: | Episodes documented by imaging techniques (ultrasound, echocardiography, pulmonary CT angiography) of venous thrombotic phenomena (including pulmonary embolism or in other territories) and arterial during the stay in the ICU and that required systemic anticoagulation at therapeutic doses will be included. Specifically, acute pulmonary embolism was defined when confirmed by chest CT angiography (segmentary or lobar or main pulmonary branch arteries) or when highly suspicious according to clinical assessment and treated accordingly by the attending physician |
| Acute viral myocarditis: | Clinical definition based on the presence of acute dilated cardiomyopathy accompanied by findings on the electrocardiogram such as nonspecific alterations at ST elevations similar to an acute coronary syndrome, associated with hemodynamic compromise or cardiogenic shock, presence of life-threatening arrhythmias (complete atrioventricular block, ventricular tachycardias ), and patients with heart failure pictures whose condition does not respond to conventional management, associated with elevated markers of myocardial damage (mainly ultrasensitive troponin I or troponin T) (2, 3). |
| Cardiovascular dysfunction | Defined as a Sequential Organ Failure Assessment Score (SOFA) score higher than two points (4). |
| NEUROMUSCULAR COMPLICATIONS |  |
| Delirium: | Acute confusion syndrome characterized by periods with variable levels of consciousness and / or alterations in behavior and / or perception of the environment, associated or not with psychomotor agitation (5). |
| Neuromuscular weakness | Weakness of the limbs and / or difficulty in “weaning” with no other causes detected except for critical illness or its treatment. It is independent of whether it is due to polyneuropathy or myopathy |
| Viral encephalitis: | It is characterized by altered state of consciousness, associated with acute fever (not explained by other concomitant bacterial infections) and accompanied by focal neurological symptoms, such as seizures, focal neurological deficits and signs such as pleocytosis in the cerebrospinal fluid (CSF), together with alterations in the tracing of the electroencephalogram (EEG) or evidence of findings of structural lesions in imaging studies (brain MRI, skull CT) (6). |
| MAIN DEATH-ASSOCIATED REASONS |  |
| Refractory hypoxemia: | Peripheral oxygen saturation measured by pulse oximetry less than 80%, or PaO2 <60 mmHg of arterial blood sample despite maximum ventilatory support (FiO2 100%, PEEP level> 5 cm H_2_O) with maintenance of MAP> 60 mmHg or SBP> 90 mmHg in the 3 hours prior to death. |
| Refractory shock: | Defined as mean arterial pressure less than 60 mmHg or systolic blood pressure less than 90 mm Hg despite vasoactive support (norepinephrine> 2 mcg / kg / min and / or dobutamine> 20 microgr / kg / min and serum lactate level > 2 mmol / L (18 mg / dL)) during the 3 hours prior to death, associated or not with organ failure, but with no evidence of documented infection in the previous 24 hours and with peripheral oxygen saturation greater than 80% oo PaO2> 60 mmHg of arterial blood sample. |
| Septic shock: | Shock that meets the criteria for refractory shock as a consequence of a documented infection in the 48 hours prior to death (7). |
| Severe arrhythmia: | Any malignant arrhythmia (ventricular or supraventricular) irreversible and causing the cessation of cardiac activity; Arrhythmias that occur in the 5 minutes prior to death are excluded |
| Acute Pulmonary thromboembolism: | Clinical suspicion, associated with any of the classifications (Wells or Geneva) according to the probability as low, intermediate or high and the determination of the high D-dimer (> 500 μg / L) in the 12 hours prior to *death*  (8). |
| Accident related to mechanical ventilation: | Episode during the period of mechanical ventilation of obstruction of the endotracheal tube or exit of the endotracheal tube that causes the accidental and irrecoverable loss of the patency of the patient's artificial airway and causes his death. |
| Ventilator-associated pneumonia (VAP): | When the physician responsible for the patient´s care includes the diagnosis or the patient receives specific antibiotics for VAP, AND with the presence of the following criteria (9):  New or worsening of Infiltrate on X-ray or Chest CT scan.  Leukocytosis / Leukopenia (white blood cell count> 12,000 cells / μl or <4,000 cells)  Purulent tracheobronchial secretions.  Fever / Hypothermia (peripheral body temperature on the corresponding day ≥ 38.5ºC or <36ºC).  Positive cultures of the tracheal aspirate (if yes, when the culture of the tracheal aspirate isolates potentially pathogenic microorganisms with significant growth (> 10^3^ CFU / uL); *Candida sp, coagulase negativ*e *staphylococcus* and normal oropharyngeal flora are excluded from the diagnosis). |

**References**

1. The ARDS Definition Task Force*, Ranieri VM, Rubenfeld GD, Thompson BT, Ferguson ND, Caldwell E, Fan E, et al. Acute Respiratory Distress Syndrome: The Berlin Definition. JAMA*2012; 307(23):2526–2533.*
2. Cooper LT Jr. Myocarditis. N Engl J Med 2009; 360 (15): 1526-38.
3. Kindermann I, Barth C, Mahfoud F, et al. Update on myocarditis. J Am Coll Cardiol 2012; 59 (9): 779-92.
4. Raith EP, Udy AA, Bailey M, McGloughlin S, MacIsaac C, Bellomo R, Pilcher DV; Australian and New Zealand Intensive Care Society (ANZICS) Centre for Outcomes and Resource Evaluation (CORE) . Prognostic accuracy of the SOFA score, SIRS criteria, and qSOFA score for in‐hospital mortality among adults with suspected infection admitted to the intensive care unit. **JAMA** 2017; 317:290–300.
5. Devlin JW, Skrobik Y, Gelinas C, et al. Clinical Practice Guidelines for the prevention and management of pain, agitation / sedation, delirium, immobility, and sleep disruption in adult patients in the ICU. Crit Care Med 2018; 46 (9): e825-e873.
6. Kramer AH. Viral encephalitis in the ICU. Crit Care Clin. 2013; 29 (3): 621-649.
7. Shankar-Hari M, Phillips GS, Levy ML, et al: Developing a new definition and assessing new clinical criteria for septic shock: for the Third International Consensus Definitions for Sepsis and Septic Shock (Sepsis-3). JAMA 2016; 315:775–787.
8. Uresandi F, Monreal M, Ferrán García-Bragadoc F, et al. National consensus on the diagnosis, risk stratification and treatment of patients with pulmonary thromboembolism. Arch Bronconeumol 2013; 49 (12): 534–547.
9. Torres A, Niederman M.S,  Chastre J, et al. International ERS/ESICM/ESCMID/ALAT guidelines for the management of hospital-acquired pneumonia and ventilator-associated pneumonia European Respiratory Journal 2017; 50: 1700582.
